# Sinking Jelly-Carbon Unveils Potential Environmental Variability along a Continental Margin

**DOI:** 10.1371/journal.pone.0082070

**Published:** 2013-12-18

**Authors:** Mario Lebrato, Juan-Carlos Molinero, Joan E. Cartes, Domingo Lloris, Frédéric Mélin, Laia Beni-Casadella

**Affiliations:** 1 Department of Biogeochemistry and Ecology, Helmholtz Centre for Ocean Research Kiel (GEOMAR), Kiel, Germany; 2 Department of Geosciences, Scripps Institution of Oceanography, San Diego, California, United States of America; 3 Institut de Ciències del Mar de Barcelona (CSIC), Barcelona, Spain; 4 Joint Research Centre, Ispra, Italy; Heriot-Watt University, United Kingdom

## Abstract

Particulate matter export fuels benthic ecosystems in continental margins and the deep sea, removing carbon from the upper ocean. Gelatinous zooplankton biomass provides a fast carbon vector that has been poorly studied. Observational data of a large-scale benthic trawling survey from 1994 to 2005 provided a unique opportunity to quantify jelly-carbon along an entire continental margin in the Mediterranean Sea and to assess potential links with biological and physical variables. Biomass depositions were sampled in shelves, slopes and canyons with peaks above 1000 carcasses per trawl, translating to standing stock values between 0.3 and 1.4 mg C m^2^ after trawling and integrating between 30,000 and 175,000 m^2^ of seabed. The benthopelagic jelly-carbon spatial distribution from the shelf to the canyons may be explained by atmospheric forcing related with NAO events and dense shelf water cascading, which are both known from the open Mediterranean. Over the decadal scale, we show that the jelly-carbon depositions temporal variability paralleled hydroclimate modifications, and that the enhanced jelly-carbon deposits are connected to a temperature-driven system where chlorophyll plays a minor role. Our results highlight the importance of gelatinous groups as indicators of large-scale ecosystem change, where jelly-carbon depositions play an important role in carbon and energy transport to benthic systems.

## Introduction

The biogenic production of organic matter and its remineralization while sinking drive the biological pump [1], [2], fuelling benthic ecosystems in continental margins and the deep ocean [3]. The process includes CO_2_ fixation and transport, which drives chemical gradients in the water column and benthopelagic couplings, which have global implications in the carbon cycle. In a changing ocean, growing evidence warns on the weakening of the biological carbon pump entailed by rising ocean temperatures and atmospheric CO_2_ [4]. This might impact marine ecosystems dynamics and resources in negative ways [5]. Recently, however, observations of fast sinking biomass of gelatinous zooplankton [Cnidaria (Scyphozoa and Hydrozoa), Thaliacea (Pyrosomida, Doliolida, Salpida) and Ctenophora] provide evidence on their potential role as a vector of carbon export (hereafter jelly-falls) [6], [7], [8].

Advancing research on jelly-falls has helped a better understanding of former studies of these events, e.g. off New Zealand shelves [9], in the mid slope of the Tasman Sea [10], in New South Wales in the shelf [11], in the deep Atlantic Ocean [12], [13], off Ivory Coast shelf and slope [7], and in the Arabian Sea deep slope [6]. During the process of jelly-biomass remineralization, dissolved organic carbon (DOC) and nutrients [14] are released, while oxygen is heavily consumed [15]. Biomass enters trophic webs consumed by bacteria or macro- and mega-fauna in benthic ecosystems [16], [7], [17] or through bacterioplankton while sinking [18], [19], [20]. Observational data of post-bloom depositions of scyphozoans and thaliaceans in continental margins [21] show that these events deliver at times more organic carbon to the seafloor than phytoplankton-based export [6], [7]. In the Atlantic and Pacific Oceans the published jelly-falls are driven by pelagic tunicates, and particularly by *Pyrosoma atlanticum* [21], which does not preclude other species from contributing to the jelly-carbon depositions. The factors driving a population crash and the onset of a jelly-fall follow population ageing [22] and cumulative negative circumstances, e.g. starvation, parasitism, infection, and predation [23]. Thus far, however, little is known on the space-time variation of jelly-carbon depositions, and the spatial scale at which the process occurs. Yet, any potential link with climate, anthropogenic forcing or their synergies is speculative.

Here we present benthopelagic evidence on interannual variations of jelly-falls associated biomass and carbon along an entire continental margin in the northwestern Mediterranean Sea. We show a significant increase in the jelly-carbon accumulation and depositions paralleling regional environmental changes. Our results shed light into post-bloom gelatinous zooplankton biomass benthopelagic couplings at large scale. Lastly, the data add important information to initially understand the magnitude and variability of jelly-carbon in the biological pump at a regional scale.

## Materials and Methods

### The MEDITS Survey Programme

The International bottom trawl survey in the Mediterranean Sea (MEDITS programme) was designed to produce scientific data on benthic and demersal species. This was carried out in seasonal bottom trawl surveys [24], including all shelves and slopes (10 to 800 m) of all Mediterranean countries. A standardized sampling method for all surveys was used to allow inter-site comparison [25]. In partner programmes such as MEDITS-ES [25] along the Iberian Peninsula, catch records of gelatinous zooplankton species such as *Pyrosoma atlanticum* (catalogued as non-commercial and bycatch) were recorded from 1994 to 2005 following an identical protocol to the commercial species. The availability of these rare data with a spatial and a temporal component opened a unique opportunity to study gelatinous biomass depositions and the associated carbon in a time-series manner, linking results to biological, hydrographic, and climate variables retrieved from the same time interval.

### The Trawl System: Sampling Strategy and Details

The trawling gear was operated in the *R/V Cornide de Saavedra* with a minimum towing power of 368 kW and 4.5 tons of bollard pull. The same vessel and crew were used every year to minimize uncertainty in sampling effort. The sampling device used in all surveys was a bottom trawl with a GOC 73 net designed for experimental fishing [26]. A special device controlled trawl geometry, resulting in a vertical opening of 2.75±0.52 m (named *a*) and a horizontal opening of 19.30±2.47 m (named *b*) (calculated for each individual haul from 1994 to 2005, *n* = 247 hauls) (Table S2, S3, S4). The cod end mesh size was 20 mm. The fishing speed was 2.89±0.23 knots (5.12±1.16 km h^−1^) on the ground (*n* = 247 hauls). Every year, the total number of hauls was between 95 and 105. Haul duration was always fixed to 30 minutes above 200 m (*n* = 81 hauls) and 60 minutes below 200 m (*n* = 166 hauls). This resulted in a temporally balanced sampling effort throughout the survey period, which makes unnecessary correcting for temporal bias and trawling effort. The resulting trawled areas (*T*_AREA_) were 47,137±5,412 m^2^ and 100,499±21,834 m^2^ depending on the depth strata (Table S2, S3, S4, for individual trawl areas and volumes). These figures were calculated assuming that the front of the GOC 73 trawl was an elliptical cylinder (named *h*):
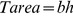
(1)

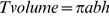
(2)where π = phi (3.1416…), *a* = vertical opening size, *b* = horizontal opening size, and h = cylinder height, which in this case is the distance travelled by the haul on the seabed.

**Figure 1:**
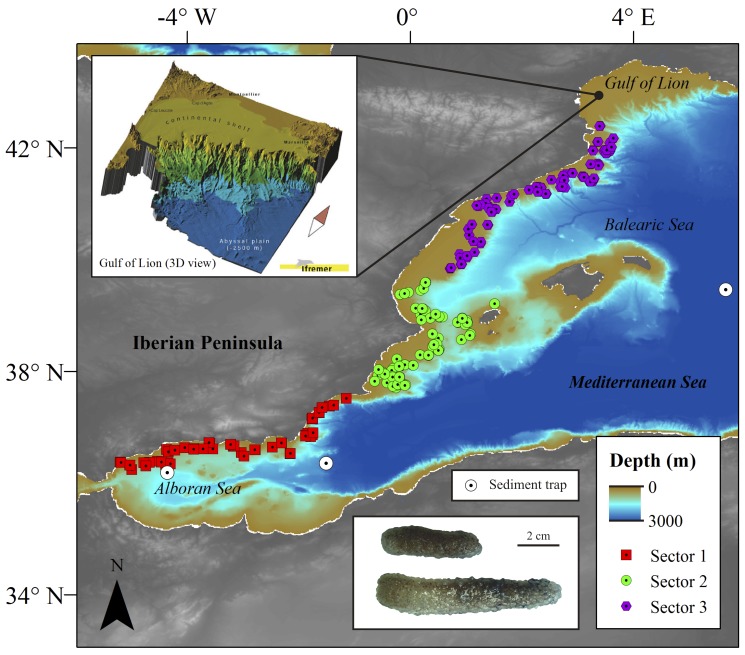
Study region in the western Mediterranean Sea continental margin. The individual sampling sites (trawls) are presented in this map separated by sector to show the large scale of the depositions. Also included is a detailed 3D bathymetry display (copyright by Ifremer) of the canyon complexity in the north of sector 3 and a photo of freshly caught biomass. General bathymetry map data taken from the General Bathymetric Chart of the Oceans (GEBCO) digital atlas.

The study area was divided into three geographical sectors to facilitate spatial and temporal data analyses (Fig. 1): Sector 1 - from Tarifa (36.00°N/5.59°W) to Cape Palos (37.62°N/0.60°W), Sector 2 - from Cape Palos (37.62°N/0.60°W) to Sagunto (39.64°N/0.19°W), and Sector 3 - from Sagunto (39.64°N/0.19°W) to Cape Creus (42.27°N/3.30°E). In brief, sector 1 included the Alboran Sea, which is an area highly influenced by Atlantic waters. This makes the sector highly variable at a mesoscale level concerning water masses circulation and primary production [27], [28]. Eddies are known to reach northwards of the Eivissa Channel [29] in Sector 2, while the Balearic Basin from Eivissa Island to the Gulf of Lions (mainly Sector 3) is characterized by the occurrence of thermohaline fronts over the shelf-slope break (also occurring in the Alboran Sea). The Balearic basin is influenced by Levantine Intermediate waters (LIW) in both the Catalan margins and the NW of the Balearic Islands [29], [30]. Sector 2 and 3 are heavily dissected by canyons, which play a major role in cascading events by transporting particles and food parcels to deeper waters and benthic systems [31].

The trawling stations were distributed following a bathymetric stratified sampling scheme with random hauls inside each stratum. The bathymetric limits used were: 10–50 m, 50–100 m, 100–200 m, 200–500 m and 500–800 m. The hauls were conducted at a constant depth never exceeding 5% of the initial depth in a straight line (Table S2, S3, S4). Each position was selected randomly in small sub-areas. Sampling was always conducted during spring and early summer, from April to July, always in the same stations. Hence, the consistency of the sampling approach makes *P. atlanticum* catches comparable through time (Table S2, S3, S4). Once onboard, the total weight (per individual) and the total number of individuals per haul was recorded. The procedure to collect any *P. atlanticum* data was as rigorous as for the commercial species.

### Ethics Statement (*Pyrosoma atlanticum* Samples)

*Pyrosoma atlanticum* carcasses were collected in 2010 by means of trawling hauls at 1226 m in the Catalan Sea for elemental chemistry analyses. Permissions to obtain the samples were granted by the Spanish Ministry via the ANTROMARE3 cruise, and project CTM2009-12214-C02-01-MAR and from the MEDITS-ES project as described in [25]. No marine protected area or protected species were sampled or affected.

### *Pyrosoma atlanticum* Biochemistry and Field Estimations

From the collected *Pyrosoma atlanticum* samples, only four carcasses were available and measured (*n* = 4, length = 5.45±0.22 cm), then frozen at −20°C and dried for 48 h at 60°C, and weighted (*n* = 4–2.092±0.065 mg dry weight). A sub-sample from each carcass was cut and re-weighted (*n* = 4–1.037±0.027 mg dry weight). The four samples were then introduced in tin vessels and closed for analysis. Organic carbon and nitrogen were measured in a Euro EA 3000 elemental analyser with acetanilide standards at the Helmholtz Centre for Ocean Research Kiel (GEOMAR) (Germany). The mean carcass organic carbon and nitrogen dry weight % were 20.08±5.41 and 3.35±0.86 respectively, while the C/N (mol/mol) was 5.12±0.15. Though we used a low sample number, our final data were within the range of published numbers [32]. These conversion factors were used to assess the organic carbon and nitrogen standing stocks based on the wet weights recorded in trawls. Wet weight conversion (wt/dwt %) were done using published data [7] from samples collected in the same area in the spring of 2007 (wt/dwt % = 12.05±1.17). Using the total number of carcasses and the total wet weight from each trawl, we computed figures of dry weight, and then organic carbon and nitrogen percentages using the values from the elemental analyzer. The trawled (*T*) biomass, POC and PON standing stocks in mg per m^2^ per haul were calculated using the individual trawl biomass (*T*_BIOMASS_) using the *T*_AREA_ from Eq. 1 (Table S2, S3, S4) along with the trawl POC (*T*_POC_) and PON (*T*_PON_) from individual hauls as follows:
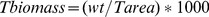
(3)

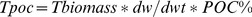
(4)

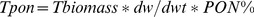
(5)where wt = wet weight, *T*_AREA_ = *b* h (*see* Eq. 1), dw/dwt % = 12.05, POC % = 20.08, and PON % = 3.35. POC and PON %s were used directly from the dry weight (elemental analyser).

### *Pyrosoma atlanticum* Sinking Speed

*Pyrosoma atlanticum* carcasses collected in 2010 were used to measure biomass sinking rates in a separate experiment published along with other gelatinous species [8]. In brief, Pyrosoma samples were collected in the Catalan Sea by plankton net (CTD field surface temperature = 24.49°C, salinity = 37.75, density = 1025.60 kg m^−3^), then frozen at −20°C and sent frozen to OceanLab, Jacobs University (Germany). Organisms were thawed for 24 h at 5°C (to prevent degradation) in a temperature-controlled room in the same water used in the column before each experimental day. There is no statistical difference between freshly sunk and thawed samples of gelatinous carcasses [8], which justifies our method. Pyrosoma were sunk in a clear acrylic column 118.50 cm tall, with a diameter of 19 cm (volume = 30 L) in a temperature-controlled enclosure at 10°C. The acrylic column was filled with freshwater, and the salinity was established by adding NaCl. Owing to the few samples available, three carcasses were sunk 3 times each under identical field density conditions, e.g. temperature = 11.50°C, salinity = 37.80, and density = 1028.89 kg m^−3^. We placed a 1 m ruler inside the column to have a dimension reference for post-video analyses. The exact water column height before each sinking run (100 cm) and sinking events were recorded with a video camera Canon Legria HF R16 mounted on an Erno P-55 tripod placed 1 m away from the column. All videos were analyzed with ImageJ software during terminal velocity on a straight line. The final averaged sinking rate the carcasses was 1278±133 m day^−1^.

### Acquisition of Temperature, Chlorophyll *a*, and Hydroclimate Data

We chose Chlorophyll *a* (Chla) as a representative of the main seawater biological variable. We assessed correlative associations in the covariance of two trophic systems, not doing a mass balance relationship between primary consumers and pyrosoma. We did not apply algorithms to convert Chla to primary production figures because most primary production in the Mediterranean occurs in deep-water layers (at 50–80 m) and satellite imagery resolution is not good at this depth. Chla data were derived from the Sea-viewing Wide Field-of-view Sensor (SeaWiFS) and the Moderate Resolution Imaging Spectroradiometer (MODIS, on-board Aqua) missions. We used as Chla (and also temperature) locations, the geographical coordinates where trawls started, assuming that pyrosoma inhabited water masses in the vicinity (search radius of ±1 km). The Chla data processing followed the methods extended to the entire Mediterranean basin. First, the remote sensing reflectance data from the 2 satellite missions were merged through an optically-based merging technique. Then, Chla was computed with two algorithms, one proposed for the open Mediterranean waters [33], [34] and one developed for coastal regions [35]. Note that in the coastal area we did not include high Chla levels of e.g. local upwelling events, riverine inputs or similar. The domain of applicability of each algorithm was determined through a novelty detection approach [36] by computing the probability of each input reflectance spectrum of belonging to the optical class associated with open sea or coastal waters. The final Chla values were a weighted average with weights defined by these probabilities. Satellite values were extracted from an equidistant grid with a 2-km spatial resolution for the location and time of interest. We divided the Chla data in two parts: 1) Specific 1-day, 8-day and 1-month composites (depending on availability) at the exact site of an actual jelly-fall from April to June, and 2) monthly composites for each year at each location. Chla data however did not cover the years 1994 to 1997, so the available data started in 1997 (Fig. S1). For temperature (Sea Surface Temperature) we used the Pathfinder data set [37] obtained from the Physical Oceanography Distributed Active Archive Center (PODAAC, NASA). The data were mapped on a 4.6-km equidistant global grid. We divided the temperature database in the same way as Chla. In this case, temperature data were available from 1994 to 2005 in 1-day composites in part 1 (thus we did not use 8-day and 1-month data), and from 1998 to 2005 in part 2. For analysis, we only used data from 1997 to 2005 to match the Chla time series (Fig. S1). For both Chla and temperature, the extracted satellite values were obtained by bi-linear interpolation of the satellite data around the location of interest (Table S3, S4). The results were considered valid only if 4 satellite retrievals were available.

Hydroclimate data were obtained from the NCEP/NCAR re-analysis of monthly series from the Earth System Research Laboratory of the National Oceanic and Atmospheric Administration (NOAA) (http://www.esrl.noaa.gov/psd/data/timeseries/). The long term trend of hydroclimatic forcing was extracted by applying a principal component analysis (PCA) on the sea surface temperature, precipitation rate, outgoing long- wave radiation and 500 mb geopotential height monthly time series. Only the first component (PC1) was used for analysis. We also used large scale climate drivers of the western Mediterranean hydroclimate conditions, e.g. the North Atlantic Oscillation (NAO) and the Northern Hemisphere Temperature anomalies (NHT), to quantify the extent to which the observed trends in jelly-biomass deposition are potentially related to large scale climate phenomena. The NAO values were retrieved from https://climatedataguide.ucar.edu/climate-data/hurrell-north-atlantic-oscillation-nao-index-station-based, while NHT were obtained from the Climatic Research Unit and Hadley Centre.

### Data Analyses

Before analyzing the hydroclimate, all data were seasonally detrended by subtracting the monthly average from each individual value. Principal component analysis (PCA) was applied to the western Mediterranean hydroclimate matrix (year * month). The first component resulting from this analysis (PC1 capturing 63% of the total variance) was subsequently used to investigate structural environmental changes. Structural changes in the western Mediterranean hydroclimate long term trend were addressed using Cumulative Sum of Ordinary Least Square residuals (CUSUM-OLS) [38]. The method is an extension of the classical CUSUM analysis using Ordinary Least Squares (OLS) residuals instead of recursive residuals. OLS residuals were obtained from fitting the PC1 scores to a linear regression with time as covariate, and CUSUM-OLS was used to calculate the empirical fluctuations. To identify significant changes in the structure of the fluctuations over time, a boundary limit was calculated using a generalized fluctuation test, establishing as the model null hypothesis that the fluctuations remained constant with a 0.95 confidence limit. The test of hypotheses explaining the jelly-biomass fluctuations was done using a General Linear Model (GLM) in a factorial mode, including temperature and Chla as predictors. This allowed assessing the individual effects and their interactions. A similar approach was used to assess a potential relationship with NAO and NHT, which shape interannual variations of the western Mediterranean environmental conditions. Analyses were conducted in Matlab Software (The MathWorks, Inc. UK) and R version 2.7.1.

## Results and Discussion

### Factors Governing the Jelly-biomass Depositions

**Figure 2:**
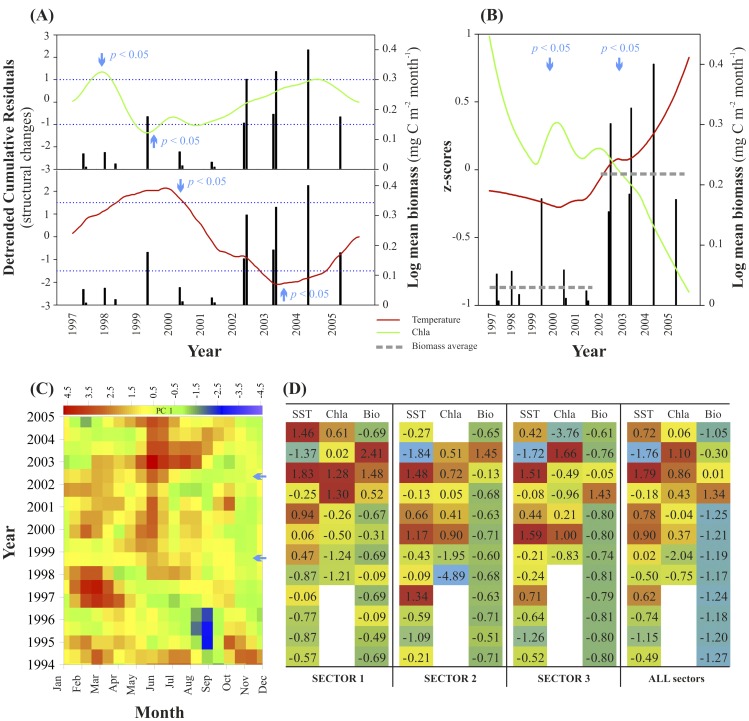
Summary of environmental and hydroclimate variables affecting the jelly-carbon depositions. (A) Structural changes of the temperature and chlorophyll a (Chla) over the period of jelly-carbon transfer. Significant temporal changes occurred in environmental predictors, although they were more evident in temperature. Significant changes are reached when the predictors surpass the threshold limits (p<0.05). (B) Synthesis of environmental and biological changes with the identification of the timing of the change (along with the significance). The horizontal dashed lines represent average biomass values for the main periods before and after 2001 (significantly different, t-test, p<0.05). (C) Monthly hydroclimate first principal Component (PC1) individual values for the study period from 1994 to 2005. (D) Quartile analysis of the zscores of temperature, Chla and the normalized (per unit area) biomass from 1994 to 2005 separately for each sector and then for all sectors.

Structural analysis of hydroclimate, temperature, and chlorophyll *a* (Chla) portrayed significant changes (structural fluctuation process test, p<0.05) that took place in the late 1990s and early 2000s (Fig. 2, S1, S2). In particular, temperature and Chla moving variances varied conspicuously in the early 2000s (Fig. S1). This is supported by the analysis of observational data showing a significant warming (Figs. 2B, S2, S3) [38] that yielded a 40% lengthening of summer conditions [39]. Such changes translated into a favorable environment (e.g. temperature-stable water masses and phytoplankton community dominated by small size-classes) [40] for the increase of *P. atlanticum* biomass, which likely enhanced jelly-carbon depositions (Figs. 1B, S2). We tested the hypothesis and assessed the contribution of environmental variables to the overall trend of accumulated jelly-biomass. The link between the aforementioned environmental changes and biomass was significant (p<0.05, Table S1), and the hypothesis decomposition translated into a temperature-driven system (p<0.05) with no statistical support for Chla, nor for a time effect, but for the combined effect of all the variables (p<0.01). In addition, the observed trend of Pyrosoma biomass deposition was significantly linked with the large-scale temperature trend indexed by the NHT as well as with the NAO (p<0.05) (Fig. S4, Table S1). The influence of these drivers, NHT and NAO, acts at several spatial scales in the western Mediterranean driving the physical environment of pelagic communities. Also, it is worth noticing that the NHT exerts a higher influence, as shown by the GLM results (Table S1), likely due to the strong NHT contribution to the regional warming trend in the Mediterranean Sea noticed in the early 2000. Hence, the model results suggest a coupling between large scale climate drivers and the organic particle deposition events on the pluriannual scale, and further agree with previous reports on fast cascading in the western Mediterranean also linked to large scale climate [31].

### Jelly-biomass Temporal Variability

**Figure 3:**
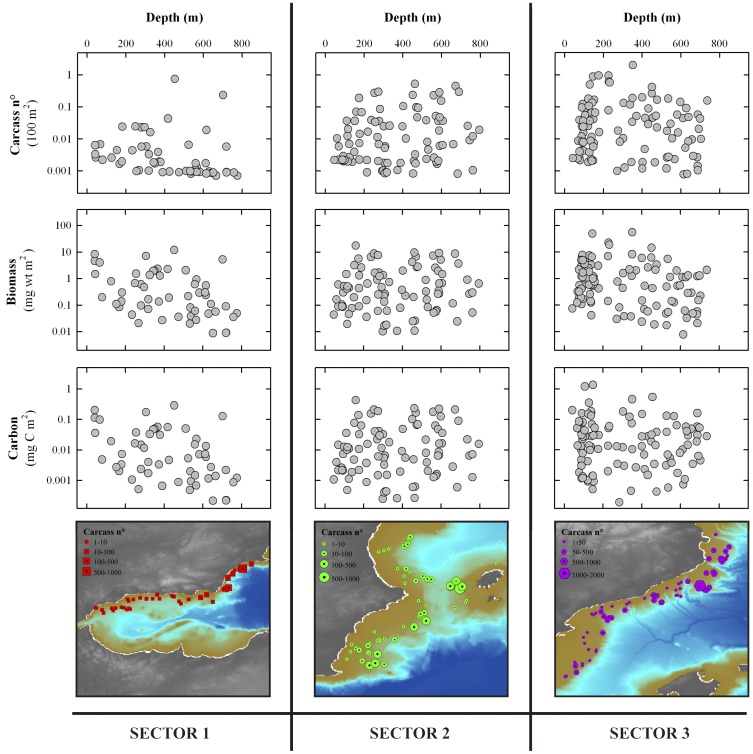
Bathymetric trends of the jelly-carbon depositions. The following data are included: *Pyrosoma atlanticum* carcass n°, total biomass and total organic carbon vs. depth normalized per unit area using the total trawled area per haul at each sector. Also included are total carcass n° raw data displayed in maps and spatial distribution per sector.

We found a significant increase in jelly-biomass depositions after 2001 that contrasted with the low values previously encountered. On average <30 carcasses per trawl were sampled except in 1996 when the number reached 471 carcasses per trawl (biomass = 49.75 mg wt m^2^) (Table S3, S4). From 2002 to 2004 a larger biomass was observed with a maxima of 1164 carcasses per trawl (biomass = 56.01 wt mg m^2^), 308 (biomass = 18.89 wt mg m^2^), and 906 (biomass = 11.98 mg wt m^2^), respectively, and total biomass varied from 1000 to >3000 g wt pyrosoma material. However, the computed total biomass (<60 mg wt m^2^) and carbon standing stock depositions (<2 mg C m^2^) (Fig. 3; Table S2, S3, S4) remained low compared to the 22 g C m^2^ observed also for pyrosoma off Ivory Coast [7]. Context sediment trap data in the open ocean near the study area (∼30 mg C m^2^ d^−1^) were higher than the jelly-carbon monthly average (Table S1), although fundamental differences in sampling approach and methodology between the two sources prevent a real comparison (Fig. 1) (see Text S1). Our study took place during spring and early summer, likely underestimating the main annual jelly-biomass deposits occurring in late summer after populations collapse [41]. Additional jelly-carbon underestimation is explained by trawling above and below 200 m, which was always conducted on 30 and 60 minutes runs, respectively (see Methods). Jelly-biomass in sectors 2 and 3 concentrated below 200 m, thus the reduced trawling time with respect to deeper strata decreased the total biomass catch (Fig. 3). Although the trawling approach provides an overall view of jelly-carbon depositions over a large area, it does not focus on any spot in particular, as a towed camera or video survey will do. These sources of uncertainty explain our concerns on jelly-carbon deposits underestimation.

Overall, jelly-carbon constitutes a sudden (e.g. days to weeks) and patchy (e.g. meters to kilometers) input of energy to benthic ecosystems, such as the deep Mediterranean, where e.g., *P. atlanticum* is the main prey of dominant fish such as *Alepocephalus rostratus* [42] at 1200–1400 m in the Balearic Basin (sector 3). Local jelly-carbon standing stock deposits reach high values (10 to 70 g C m^2^), e.g. in the Arabian Sea [6] and off Ivory Coast [7], although generally lower values in the Mediterranean Sea were expected due to the lower biological production and a totally different sampling approach. In this regard, food consumption estimations of midslope megafauna (700 m) in the survey area are 0.059 g C m^−2^ yr^−1^, which is equivalent to computed values for production by benthic and suprabenthic macrofauna [43]. The jelly-biomass was evenly distributed over the range 0–800 m depth except in sector 1 (Alboran Sea), where its accumulation decreased with depth (Fig. 3). This suggests that Pyrosoma biomass reaches shelf and slope depths within a short time frame after blooms collapse following the biomass high sinking speed (>1200 m day^−1^), which might yield very low remineralization rates in the water column that translates into a complete benthic degradation process [44]. Once the biomass reaches the seabed it is transported at varying rates down slope, accumulating along geomorphological bottom features (e.g. canyons) and creating patchiness.

### Jelly-biomass Spatial Variability

The southern Gulf of Lyon (sector 3) is characterized by extensive continental shelves (60–120 km wide), and a narrow shelf in the central coast of Catalonia. The shelf is dissected by large canyons that extend down to the Catalan margins (Fig. 1) and to the Ebro slope (also sector 2). Large biomass accumulation below 200 m indicates initial sinking in the shelf margin (Fig. 3) and a progressively benthic transport downcanyon to slope depths. We observed that the largest biomass accumulations occurred at the edge of the shelf and in canyon mouths. A similar phenomenon was noticed for phytodetritus, with larger depositions in the slope off Catalonia (with canyons) than along insular margins (with fewer canyons) [45]. This may be explained by climate-driven advection, e.g. NAO variations (Fig. S4) [46], of food from upper layers to the open sea macrobenthic communities. In turn, such variations do govern oscillations in the plankton/benthos resources available for the continental margin trophic webs. In addition, this process is linked to the formation of dense cold water cascading episodic events reaching great depths [47]. Climatic oscillations, change the rainfall regime, and together with dense shelf water cascading [31], [46] are key process in the Mediterranean to understand benthic transport of jelly-carbon. During winter, cooling and evaporation of the surface waters by northern winds pushes coastal water masses offshore. Then, surface layers increase in density up to a point where sinking starts reaching a depth where water is at equilibrium. Under conditions of high density, the surface waters sink and reach the seafloor. Cascading episodes are generally associated with winter and spring conditions, coinciding with the start of phytoplankton and gelatinous zooplankton blooms. This happens in particular in the Gulf of Lyon, corresponding to our upper trawling area (sector 3). When such dense surface water sinks and reaches the shelf seabed it moves over the bottom to the shelf edge creating a turbulent, thick and particle-loaded flow to ultimately cascade downslope along submarine canyons (up to 1 ms^−1^) [31]. The spatial distribution of jelly-biomass from the shelves to the canyons interior may be explained by the benthic current flows, which display a decreasing transport capacity, translating into particle size sorting. Speed attenuation with increasing distance from shore and depth downcanyon causes a selective deposition of particles along the canyon floor according to individual size. In our surveys, the jelly-biomass particles (large parcels) accumulated in the canyon heads, supporting this bottom current speed attenuation hypothesis.

It is worth noticing that the correlation between jelly-biomass and environmental conditions was non-stationary and varied depending on the sector. The Alboran Sea (sector 1) was characterized by turbulent mixing governed by the opposing flows of the Mediterranean and the Atlantic which enhance productivity year round (Fig. S5). High Chla levels remained through the year following eddy features, water exchange regimes, and frontal instabilities causing vertical mixing that bring cold and nutrient-rich waters to the surface [27], [48] (Fig. S5). The main Chla and pyrosome production areas are located inshore the front (at ca. 20 km from coast) [49], which explains the higher biomass in the shelf in sector 1. Early diatom blooms appear as soon as January while the late bloom is in April [50], which coincides with the period when pyrosoma blooms likely collapse (March to April), following the Chla seasonal peak (in all sectors).

In sector 2, Chla remained much stable year-round following seasonal oscillations (Fig. S5), while in sector 3, localized upwelling events in the Gulf of Lions and the influence of the Ebro Delta yielded large variability of monthly Chla (Fig. S5). This increased the jelly-carbon depositions locally following upwelling-induced pyrosoma blooms collapse. Chlorophyll concentrates in the first 50 km from shore [51], suggesting that pyrosoma bloom and collapse in the area and then quickly sink to the seabed. Isolated upwelling events occur, related to the shelf features [52]. These events might explain the complexity and patchiness of pyrosoma depositions particularly in the upper part of sector 3 (Figs. 2, 3).

### Hints for Expanding Jelly-carbon Knowledge in the Biological Pump

Our results suggest that the increased jelly-biomass deposits after 2000 are likely part of an ecosystem-wide modification in the western Mediterranean where the jelly-falls benthopelagic coupling have been naturally enhanced. However, jelly-carbon deposition is a non-stationary phenomenon coupled to productivity regimes and hydroclimate (Figure 1, S2, S3). Gelatinous zooplankton biomass oscillates over long decadal time periods [53], and our results coincide with a maxima reported in a recent global jelly-biomass trends analysis [53]. This adds positive evidence to the long-term oscillation hypothesis at a regional scale also observed on a 30 years dataset on salps in the Tasman Sea [54]. Whether jelly-carbon deposits continue to increase in magnitude and frequency in the future remains speculative, though evidence in the Mediterranean Sea shows an upward trend of jelly-biomass in the last thirty years. Our results provide initial data to further understand the magnitude and variability of jelly-carbon in continental margins. The whole Mediterranean Sea has some 518 submarine canyons [55], which translates into a near 9% of the world canyons. The gelatinous zooplankton intense blooming activity on its margins guarantees the presence of sinking jelly-carbon. The Mediterranean and any other continental margin dissected by canyons may play an active and key role in the transport and remineralization of jelly-carbon following cascading processes, which are ultimately governed by large scale hydroclimate forcing (e.g. NAO).

## Supporting Information

Figure S1 **Temporal variability in environmental variables.** z-scores and moving variance (MV) of monthly temperature and chlorophyll *a* (Chla) from 1994 to 2005 divided per sector.
(TIF)

Figure S2 **Structural changes of the environmental variables.** Changes in the temperature and chlorophyll *a* (Chla) over the period of biomass records correlating the temperature and the Chla z-scores over the biomass time-series. The Chla symbols represent annual values and the size of the symbol is scaled with the value. The two identified periods for the time of the change are also labelled along with the significance.
(PNG)

Figure S3 **Extended Principal Component Analysis.** Monthly hydroclimate first principal Component (PC1) individual values extended from 1950 to 2011 and then amplified as cumulative sum data for the study period from 1994 to 2005.
(TIF)

Figure S4 **North Atlantic Oscillation (NAO) and the Northern Hemisphere Temperature anomalies (NHT) analyses.** z-scores of Pyrosoma biomass depositions covariation with the NAO and the NHT using the data from 1994 to 2005.
(TIF)

Figure S5 **Description of the main environmental variables.** Temperature and chlorophyll *a* (Chla) mean monthly values from 1994 to 2005 at each sector. Standard deviations show the time-series variability per month.
(TIF)

Table S1 **Statistical analyses.** Details of the General Linear Model (GLM) run to test (a) time, temperature and Chla effect, and (b) North Atlantic Oscillation (NAO) and Northern Hemisphere Temperature anomalies (NHT) effect on the Pyrosma biomass over time.
(DOC)

Table S2 **Particle and jelly-carbon export comparison.** A selection of carbon deposition measurements in the Mediterranean Sea (sediment trap, and jelly-carbon in this study).
(DOC)

Table S3 **Trawling data summary.** MEDITS-ES trawling catches of *Pyrosoma atlanticum* carcasses from 1994 to 2005.
(DOC)

Table S4 **Trawling raw data.** Complete MEDITS-ES trawling catches of *Pyrosoma atlanticum carcasses* from 1994 to 2005 with additional meta-data and calculations.
(DOC)

Text S1 **Supplementary text about the methodology used.** Discussion of the study caveats and comparisons with sediment trap data.
(DOC)
