# Supplementary figures and images for: Sinking Jelly-Carbon Unveils Potential Environmental Variability along a Continental Margin
Source: PLoS One. 2013 Dec 18;8(12):e82070. doi: 10.1371/journal.pone.0082070 (PMC3867349; doi:10.1371/journal.pone.0082070)

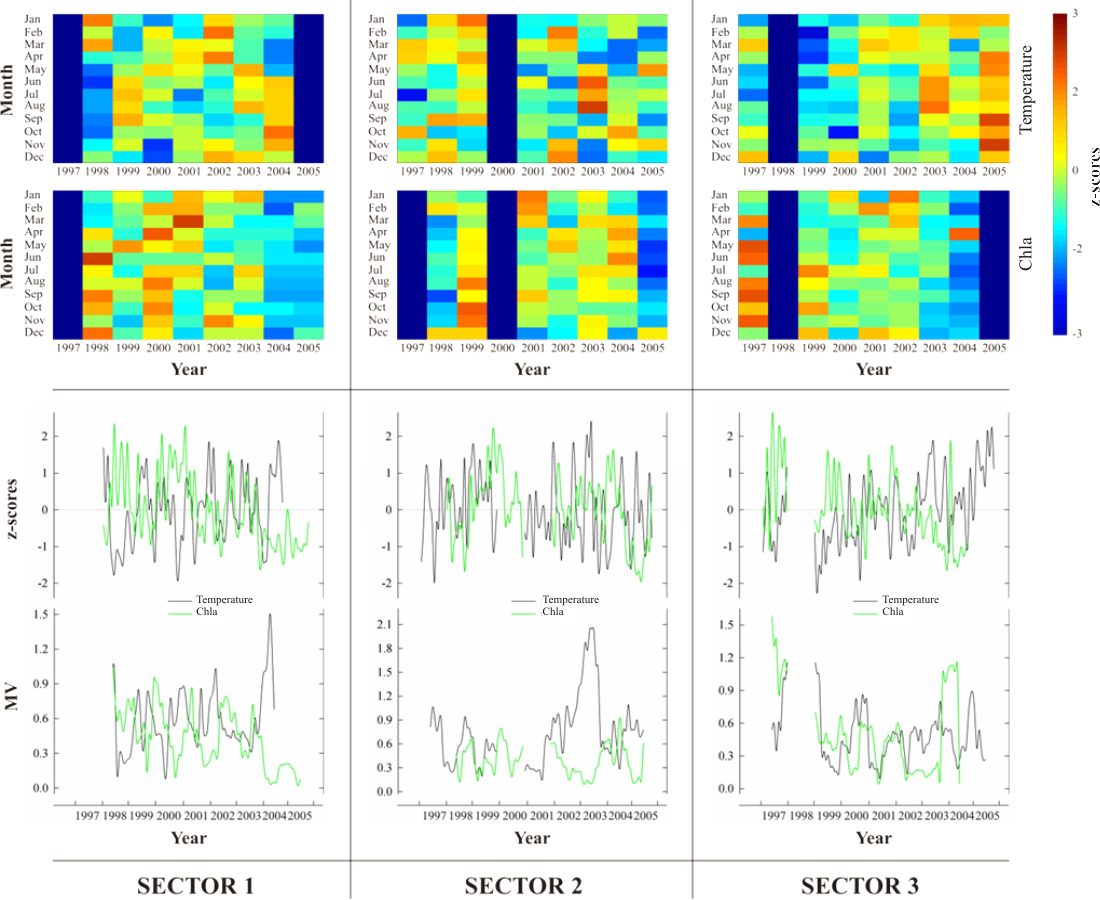

Supplement: Figure S1 — Temporal variability in environmental variables. z-scores and moving variance (MV) of monthly temperature and chlorophyll a (Chla) from 1994 to 2005 divided per sector. (TIF) [file pone.0082070.s001.tif]

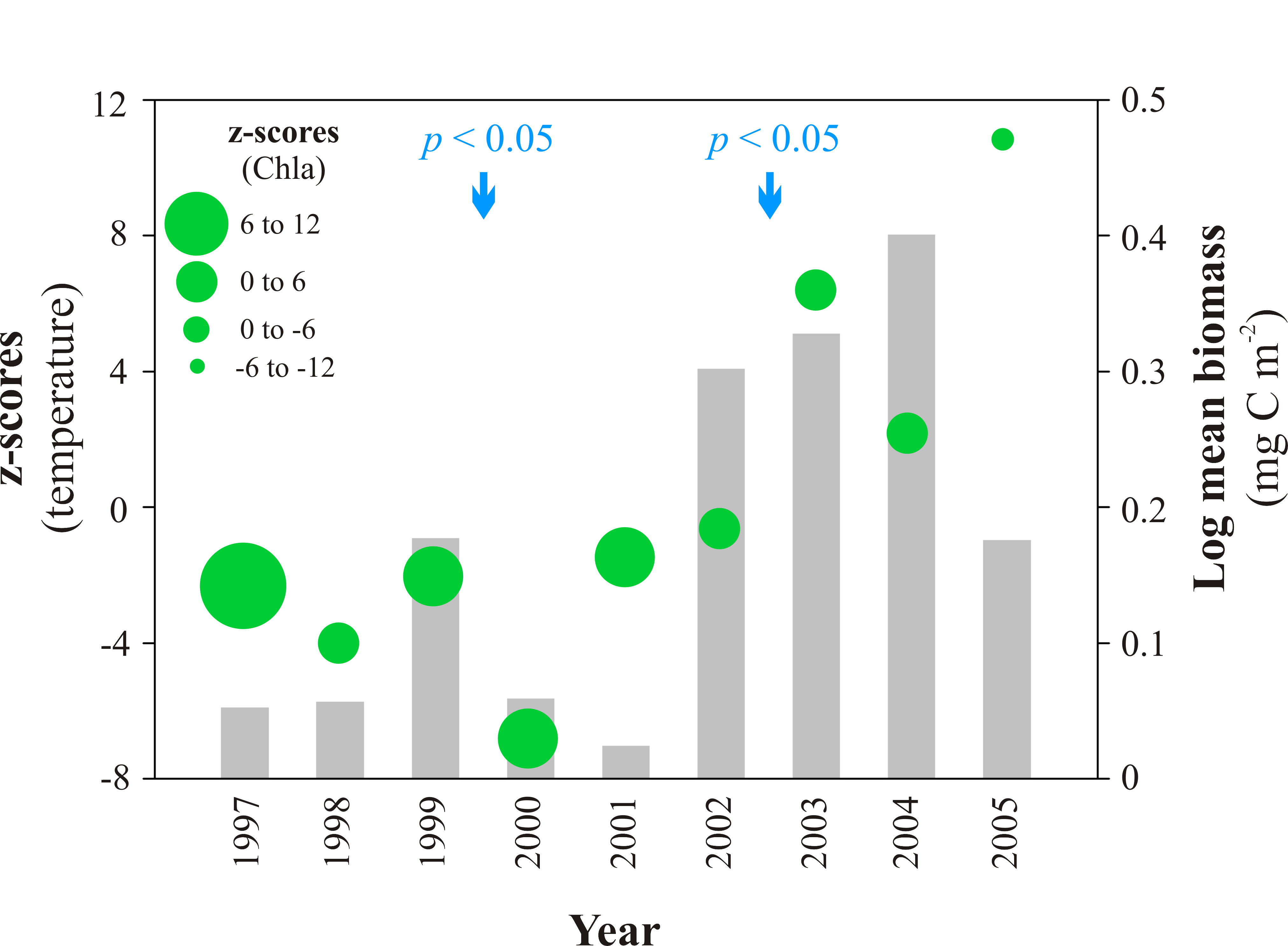

Supplement: Figure S2 — Structural changes of the environmental variables. Changes in the temperature and chlorophyll a (Chla) over the period of biomass records correlating the temperature and the Chla z-scores over the biomass time-series. The Chla symbols represent annual values and the size of the symbol is scaled with the value. The two identified periods for the time of the change are also labelled along with the significance. (PNG) [file pone.0082070.s002.png]

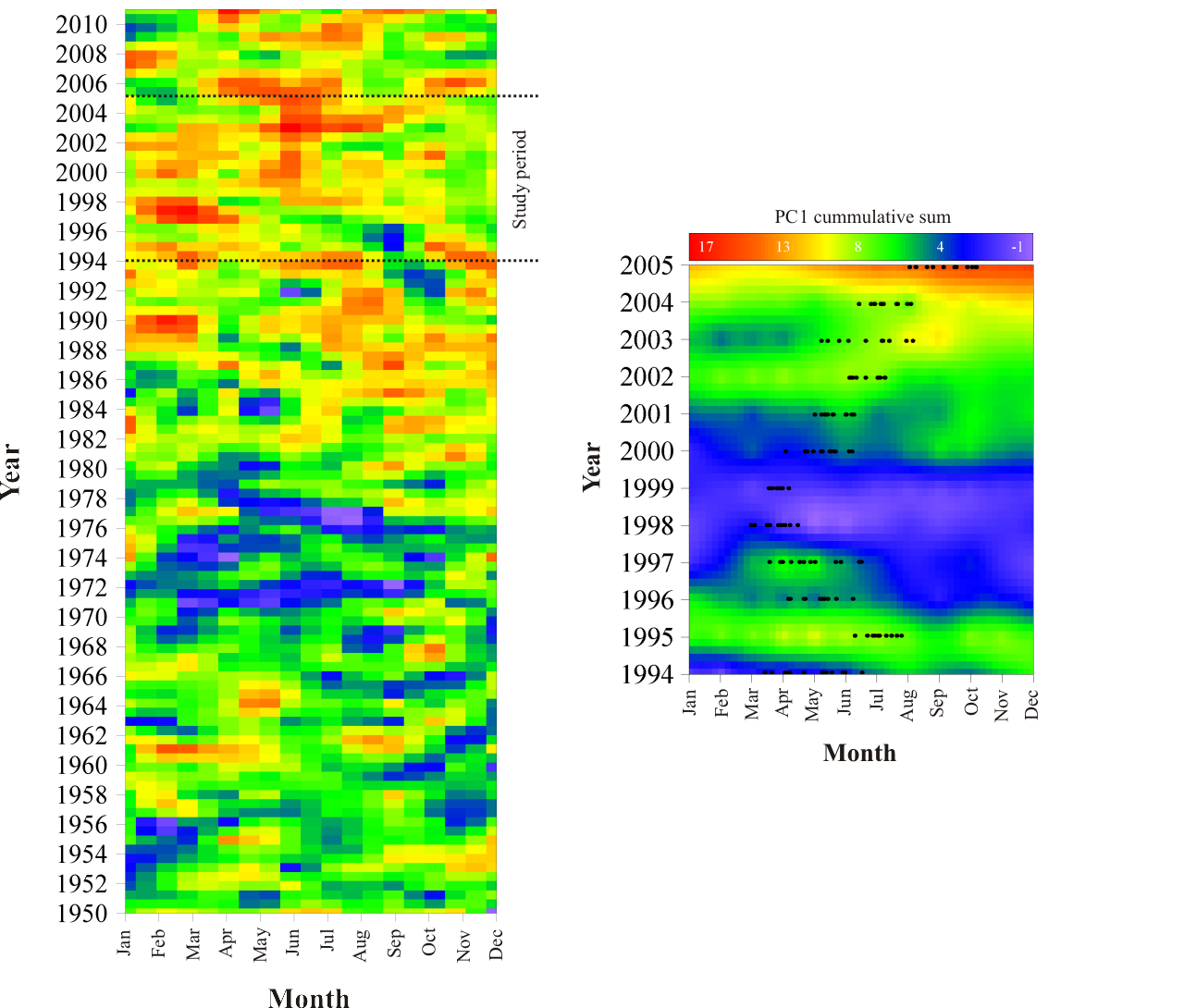

Supplement: Figure S3 — Extended Principal Component Analysis. Monthly hydroclimate first principal Component (PC1) individual values extended from 1950 to 2011 and then amplified as cumulative sum data for the study period from 1994 to 2005. (TIF) [file pone.0082070.s003.tif]

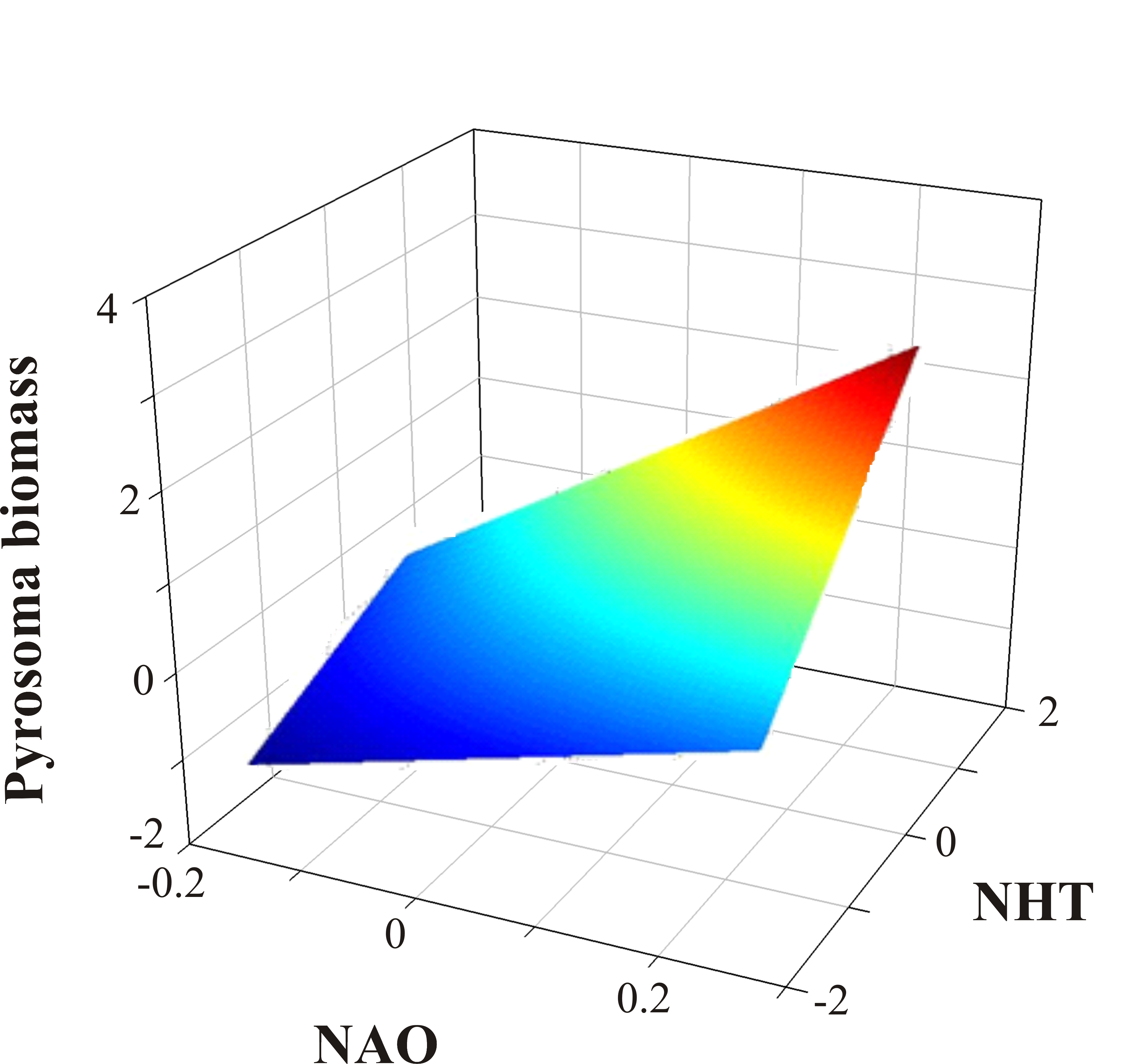

Supplement: Figure S4 — North Atlantic Oscillation (NAO) and the Northern Hemisphere Temperature anomalies (NHT) analyses. z-scores of Pyrosoma biomass depositions covariation with the NAO and the NHT using the data from 1994 to 2005. (TIF) [file pone.0082070.s004.tif]

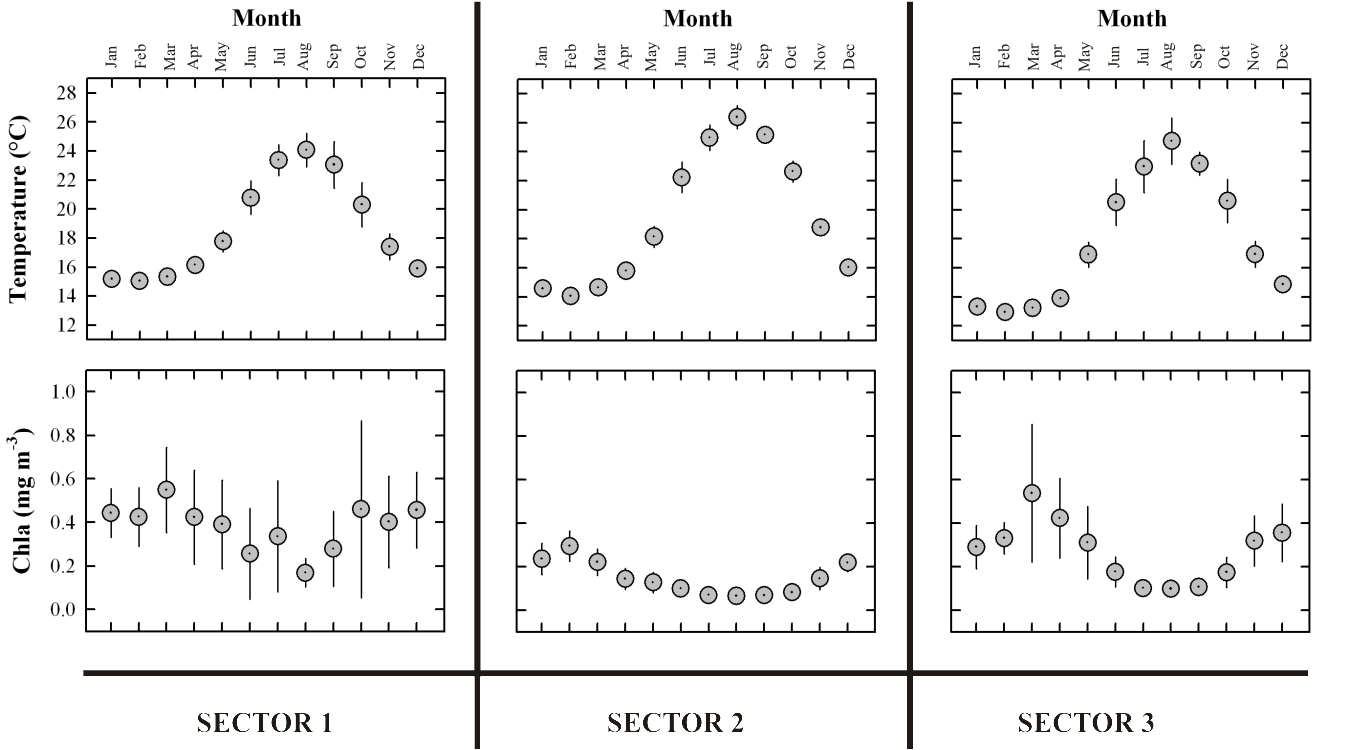

Supplement: Figure S5 — Description of the main environmental variables. Temperature and chlorophyll a (Chla) mean monthly values from 1994 to 2005 at each sector. Standard deviations show the time-series variability per month. (TIF) [file pone.0082070.s005.tif]
