# Supplementary material for: Sinking Jelly-Carbon Unveils Potential Environmental Variability along a Continental Margin
Source: PLoS One. 2013 Dec 18;8(12):e82070. doi: 10.1371/journal.pone.0082070 (PMC3867349; doi:10.1371/journal.pone.0082070)
Supplement: Table S1 — Statistical analyses. Details of the General Linear Model (GLM) run to test (a) time, temperature and Chla effect, and (b) North Atlantic Oscillation (NAO) and Northern Hemisphere Temperature anomalies (NHT) effect on the Pyrosma biomass over time. (DOC) [file pone.0082070.s006.doc]

**Table S1**

| **Effective Hypothesis Decomposition** | | | | | | |
| --- | --- | --- | --- | --- | --- | --- |
| 1. Time, Chla, Temperature, Chla*Temperature, Time*Chla*Temperature | | | | | | |
|  | SS | DF | MS | F | **p** |  |
| **Intercept** | 1.23 | 1.00 | 1.23 | 15.78 | **0.00** |  |
| **Time** | 1.00 | 1.00 | 1.04 | 1.10 | 0.29 |  |
| **Chla** | 0.24 | 1.00 | 0.24 | 3.07 | **0.08** |  |
| **Temperature** | 1.51 | 1.00 | 1.51 | 19.25 | **0.00** |  |
| **Chla*Temperature** | 0.16 | 1.00 | 0.16 | 2.11 | 0.15 |  |
| **Time*Chla*Temperature** | 7.10 | 1.00 | 7.11 | 7.84 | **0.01** |  |
| **Error** | 8.13 | 104.00 | 0.08 |  |  |  |
|  |  |  |  |  |  |  |
| R | R2 | SS Model | DF model | MS model | F | **p** |
| 0.46 | 0.21 | 22.7 | 14.0 | 1.62 | 1.79 | **0.05** |
| 1. NAO, NHT, NAO*NHT | | | | | | |
|  | SS | DF | MS | F | **p** |  |
| **Intercept** | 0.11 | 1.00 | 0.11 | 0.23 | 0.65 |  |
| **NAO** | 3.76 | 1.00 | 3.76 | 7.71 | **0.04** |  |
| **Temperature** | 4.41 | 1.00 | 4.41 | 9.07 | **0.02** |  |
| **NAO*Temp** | 0.48 | 1.00 | 0.48 | 0.98 | 0.37 |  |
| **Error** | 2.44 | 5.00 | 0.49 |  |  |  |
|  |  |  |  |  |  |  |
| R | R2 | SS Model | DF model | MS model | F | **p** |
| 0.80 | 0.64 | 5.08 | 2.00 | 2.54 | 5.23 | **0.05** |
