# Supplementary material for: Sinking Jelly-Carbon Unveils Potential Environmental Variability along a Continental Margin
Source: PLoS One. 2013 Dec 18;8(12):e82070. doi: 10.1371/journal.pone.0082070 (PMC3867349; doi:10.1371/journal.pone.0082070)
Supplement: Table S2 — Particle and jelly-carbon export comparison. A selection of carbon deposition measurements in the Mediterranean Sea (sediment trap, and jelly-carbon in this study). (DOC) [file pone.0082070.s007.doc]

**Table S2**

| **Carbon deposition** | **Region** | **Lat.** | **Long.** | **Depth (m)** | **Organic C deposition** | **Time component** | **Reference** |
| --- | --- | --- | --- | --- | --- | --- | --- |
|  |  |  |  |  |  |  |  |
| Sediment trap (ALB-1) | Alboran Sea | 36.23 °N | 4.25 °W | 471 | mean = 26.90 mg C m^-2^ day^-1^ | June 1997 to May 1998 | 1 |
| Sediment trap (ALB-2) | Alboran Sea | 36.01 °N | 4.29 °W | 396 | mean = 46.63 mg C m^-2^ day^-1^ | June 1997 to May 1998 | 1 |
| Sediment trap (ALB-4) | Alboran Sea | 36.20 °N | 1.50 °W | 645 | mean = 26.42 mg C m^-2^ day^-1^ | June 1997 to May 1998 | 2 |
| Sediment trap (SPM) | Algero-Balearic Basin | 39.00 °N | 6.00 °E | 845 | mean = 26.02 mg C m^-2^ day^-1^ | April 2001 to May 2002 | 3 |
| Jelly-carbon | Alboran Sea - Sector 1 | 36.00 °N  37.62 °N | 5.50 °W  0.60 °W | 41 to 775  (mean = 416) | mean = 0.03 mg C m^-2^ month^-1^  (max. = 0.29) | April/May/June 1994 to 2005 | This study |
| Jelly-carbon | Balearic Sea - Sector 2 | 37.62 °N  39.64 °N | 0.60 °W  0.19 °W | 45 to 794  (mean = 366) | mean = 0.04 mg C m^-2^ month^-1^  (max. = 0.42) | April/May/June 1994 to 2005 | This study |
| Jelly-carbon | Catalan Sea - Sextor 3 | 39.64 °N  42.27 °N | 0.19 °W  3.34 °E | 42 to 736  (mean = 319) | mean = 0.07 mg C m^-2^ month^-1^  (max. = 1.35) | April/May/June 1994 to 2005 | This study |

1. Fabres J, Calafat A, Sanchez-Vidal A, Canals M, et al. (2002) Composition and spatio-temporal variability of particle fluxes in the Western Alboran Gyre, Mediterranean Sea. J Mar Sys 33-34: 431-456.

2. Sanchez-Vidal A, Calafat A, Canals M, Fabres, J (2004) Particle fluxes in the Almeria-Oran Front:control by coastal upwelling and sea surface circulation. J Mar Sys 52: 89-106.

3. Zuñiga D, et al. (2007) Particulate organic carbon budget in the open Algero-Balearic Basin (Western Mediterranean): Assessment from a one-year sediment trap experiment. Deep Sea Res I 54: 1530-1548.
