# Supplementary material for: Sinking Jelly-Carbon Unveils Potential Environmental Variability along a Continental Margin
Source: PLoS One. 2013 Dec 18;8(12):e82070. doi: 10.1371/journal.pone.0082070 (PMC3867349; doi:10.1371/journal.pone.0082070)
Supplement: Table S3 — Trawling data summary. MEDITS-ES trawling catches of Pyrosoma atlanticum carcasses from 1994 to 2005. (DOC) [file pone.0082070.s008.doc]

**Table S3**

| **Year/Month** | **Latitude**  Start  End | **Longitude**  Start  End | **Depth**  (m)  (Min-Max) | **Trawl speed**  (knots) | ***h***  (m)  (Min-Max) | ***T*_AREA_**  (m^2^)  (Min-Max) | ***T*_VOLUME_**  (m^3^)  (Min-Max) | **Carcass**  (n°)  (Min-Max) | **Wet wt**  (gr)  (Min-Max) | ***T*_BIOMASS_**  (mg wt m^2^)  (Min-Max) | ***T*_POC_**  (mg C m^2^)  (Min-Max) | ***T*_PON_**  (mg C m^2^)  (Min-Max) |
| --- | --- | --- | --- | --- | --- | --- | --- | --- | --- | --- | --- | --- |
| 1994/June | 41.9443 °N  41.9757 °N | 3.5512 °E  3.5928 °E | 564-690 | 2.80 ± 0.84 | 4015-6348 | 86724-139656 | 980825-1228477 | 2-2 | 8-11 | 0.050-0.120 | 0.001-0.003 | 0.000-0.001 |
| 1995/April - May | 36.6137 °N  40.8718 °N | 0.2413 °W  4.3492 °W | 122-591 | 2.80 ± 0.00 | 2575-5395 | 38764-121927 | 231383-900939 | 1-12 | 4-96 | 0.048-0.915 | 0.001-0.022 | 0.000-0.004 |
| 1996/May | 36.3230 °N  41.9847 °N | 0.1297 °W  4.4587 °W | 73-780 | 2.92 ± 0.20 | 2673-6514 | 37460-141053 | 170765-1203302 | 1-471 | 1-2500 | 0.020-49.75 | 0.001-1.204 | 0.000-0.201 |
| 1997/May - June | 38.8393 °N  42.3940 °N | 0.4885 °E  3.5417 °E | 134-692 | 2.65 ± 0.19 | 2650-5002 | 47700-103371 | 534121-1215085 | 1-18 | 2-51 | 0.021-0.536 | 0.001-0.013 | 0.000-0.002 |
| 1998/May | 36.2770 °N  38.4723 °N | 0.4687 °E  5.0687 °W | 311-671 | 2.81 ± 0.53 | 4062-6968 | 77418-140057 | 340502-1027780 | 1-5 | 8-242 | 0.057-2.349 | 0.001-0.057 | 0.001-0.009 |
| 1999/May | 36.8240 °N  42.1233 °N | 0.4943 °W  3.3762 °E | 92-295 | 3.00 ± 0.39 | 2323-5786 | 34380-100098 | 911952-864252 | 1-1 | 2-84 | 0.087-1.589 | 0.001-0.038 | 0.000-0.006 |
| 2000/May - June | 36.3435 °N  42.4005 °N | 1.7772 °W  5.2102 °W | 41-428 | 2.75 ± 0.10 | 2496-5224 | 42731-104717 | 410314-1008333 | 1-2 | 3-56 | 0.030-1.476 | 0.001-0.036 | 0.000-0.006 |
| 2001/May - June | 36.2612 °N  41.4673 °N | 0.2453 °W  4.9417 °W | 173-750 | 2.75 ± 0.14 | 2603-5777 | 44251-128369 | 816546-1375411 | 1-24 | 1-35 | 0.010-0.380 | 0.000-0.009 | 0.000-0.002 |
| 2002/May - June | 36.3503 °N  41.9795 °N | 0.1488 °W  5.2088 °W | 34-770 | 2.90 ± 0.25 | 2404-5591 | 30050-132086 | 330417-1059819 | 1-1164 | 1-3200 | 0.011-56.01 | 0.001-1.355 | 0.000-0.226 |
| 2003/April - May | 36.2820 °N  42.3935 °N | 0.0037 °W  5.2080 °W | 35-774 | 2.89 ± 0.23 | 1846-7792 | 33412-175320 | 290857-1432037 | 1-308 | 1-1000 | 0.009-18.890 | 0.000-0.457 | 0.000-0.076 |
| 2004/May - June | 36.5042 °N  42.1297 °N | 0.0053 °W  3.8338 °W | 59-724 | 2.97 ± 0.09 | 2646-5763 | 34398-121022 | 216128-1135494 | 1-906 | 2-1450 | 0.020-11.981 | 0.000-0.290 | 0.000-0.048 |
| 2005/May | 37.7958 °N  41.2802 °N | 0.6240 °W  2.7827 °E | 44-636 | 2.97 ± 0.05 | 2704-5549 | 41912-123743 | 368677-971872 | 1-10 | 2-206 | 0.032-4.915 | 0.001-0.119 | 0.000-0.020 |
