# Supplementary material for: Sinking Jelly-Carbon Unveils Potential Environmental Variability along a Continental Margin
Source: PLoS One. 2013 Dec 18;8(12):e82070. doi: 10.1371/journal.pone.0082070 (PMC3867349; doi:10.1371/journal.pone.0082070)
Supplement: Table S4 — Trawling raw data. Complete MEDITS-ES trawling catches of Pyrosoma atlanticum carcasses from 1994 to 2005 with additional meta-data and calculations. (DOC) [file pone.0082070.s009.doc]

**Table S4**

| **Cast**  (n°) | **Date**  (trawl) | **Time**  Start / End | | **Latitude**  (Start / End) | | **Longitude**  (Start / End) | | **Depth (m)**  (Start / End) | | **Trawl speed**  (knots) | ***a***  (m) | ***b***  (m) | ***h***  (m) | ***T*_AREA_**  (m^2^) | ***T*_VOLUME_**  (m^3^) | **Carcass**  (n°) | **Wet wt**  (gr) | ***T*_BIOMASS_**  (mg wt m^2^) | ***T*_POC_**  (mg C m^2^) | ***T*_PON_**  (mg C m^2^) |
| --- | --- | --- | --- | --- | --- | --- | --- | --- | --- | --- | --- | --- | --- | --- | --- | --- | --- | --- | --- | --- |
| 81 | 19.06.1994 | 15.18 | 15.18 | 41.976°N | 41.932°N | 3.551°E | 3.554°E | 570 | 564 | 2.2 | 3.6 | 21.6 | 4015 | 86724 | 980825 | 2 | 11 | 0.127 | 0.003 | 0.001 |
| 82 | 19.06.1994 | 17.35 | 17.35 | 41.944 | 41.912 | 3.593°E | 3.530°E | 682 | 690 | 3.4 | 2.8 | 22.0 | 6348 | 139656 | 1228477 | 2 | 8 | 0.057 | 0.001 | 0.000 |
|  |  |  |  |  |  |  |  |  |  |  |  |  |  |  |  |  |  |  |  |  |
| 2 | 22.04.1995 | 11.30 | 11.30 | 36.614 | 36.598 | 4.349°W | 4.371°W | 122 | 130 | 2.9 | 1.9 | 14.7 | 2637 | 38764 | 231383 | 1 | 31 | 0.800 | 0.019 | 0.003 |
| 30 | 30.04.1995 | 8.50 | 8.50 | 37.748 | 37.701 | 0.241°W | 0.255°W | 387 | 390 | 2.9 | 2.2 | 20.9 | 5392 | 112693 | 778876 | 2 | 9 | 0.080 | 0.002 | 0.000 |
| 36 | 01.05.1995 | 12.33 | 12.33 | 38.027 | 38.048 | 0.322°W | 0.306°W | 139 | 139 | 2.9 | 1.9 | 19.0 | 2740 | 52060 | 310747 | 1 | 4 | 0.077 | 0.002 | 0.000 |
| 38 | 02.05.1995 | 6.24 | 6.24 | 38.354 | 38.395 | 0.487°E | 0.520°E | 577 | 576 | 2.9 | 2.1 | 22.6 | 5395 | 121927 | 804394 | 2 | 8 | 0.066 | 0.002 | 0.000 |
| 39 | 02.05.1995 | 8.46 | 8.46 | 38.473 | 38.451 | 0.424°E | 0.413°E | 171 | 180 | 2.9 | 2.1 | 20.2 | 2697 | 54479 | 359419 | 4 | 24 | 0.441 | 0.011 | 0.002 |
| 48 | 04.05.1995 | 13.05 | 13.05 | 38.484 | 38.440 | 0.477°E | 0.450°E | 310 | 309 | 2.9 | 2.3 | 18.9 | 5389 | 101852 | 735948 | 6 | 27 | 0.265 | 0.006 | 0.001 |
| 50 | 05.05.1995 | 6.25 | 6.25 | 38.863 | 38.908 | 1.026°E | 1.012°E | 269 | 269 | 2.8 | 2.6 | 20.6 | 5091 | 104875 | 856630 | 12 | 96 | 0.915 | 0.022 | 0.004 |
| 51 | 05.05.1995 | 8.28 | 8.28 | 38.892 | 38.850 | 0.969°E | 0.980°E | 453 | 461 | 2.6 | 2.9 | 21.0 | 4709 | 98889 | 900939 | 2 | 20 | 0.202 | 0.005 | 0.001 |
| 53 | 05.05.1995 | 14.17 | 14.17 | 38.894 | 38.943 | 0.921°E | 0.933°E | 591 | 589 | 3.0 | 2.1 | 20.8 | 5561 | 115669 | 763106 | 7 | 32 | 0.277 | 0.007 | 0.001 |
| 54 | 06.05.1995 | 6.33 | 6.33 | 38.690 | 38.667 | 1.084°E | 1.084°E | 124 | 124 | 2.8 | 2.4 | 14.4 | 2575 | 37080 | 279576 | 1 | 17 | 0.458 | 0.011 | 0.002 |
| 55 | 06.05.1995 | 10.37 | 10.37 | 38.529 | 38.566 | 0.956°E | 0.920°E | 523 | 520 | 2.9 | 2.3 | 16.2 | 5226 | 84661 | 611733 | 2 | 28 | 0.331 | 0.008 | 0.001 |
| 70 | 11.05.1995 | 7.21 | 7.21 | 40.666 | 40.620 | 1.403°E | 1.394°E | 406 | 398 | 2.8 | 2.3 | 21.0 | 5113 | 107373 | 775840 | 2 | 16 | 0.149 | 0.004 | 0.001 |
| 71 | 11.05.1995 | 11.15 | 11.15 | 40.872 | 40.903 | 1.449°E | 1.496°E | 529 | 521 | 2.8 | 2.3 | 16.1 | 5205 | 83801 | 605514 | 1 | 4 | 0.048 | 0.001 | 0.000 |
|  |  |  |  |  |  |  |  |  |  |  |  |  |  |  |  |  |  |  |  |  |
| 12 | 04.05.1996 | 14.00 | 14.00 | 36.323 | 36.325 | 4.459°W | 4.389°W | 780 | 771 | 3.4 | 2.7 | 22.5 | 6269 | 141053 | 1196449 | 1 | 7 | 0.050 | 0.001 | 0.000 |
| 18 | 06.05.1996 | 8.37 | 8.37 | 36.617 | 36.610 | 3.814°W | 3.876°W | 530 | 540 | 3.1 | 2.3 | 21.8 | 5616 | 122429 | 884629 | 1 | 10 | 0.082 | 0.002 | 0.000 |
| 20 | 06.05.1996 | 13.58 | 13.58 | 36.661 | 36.658 | 3.230°W | 3.175°W | 624 | 634 | 2.7 | 2.7 | 21.3 | 4914 | 104668 | 887826 | 1 | 5 | 0.048 | 0.001 | 0.000 |
| 24 | 07.05.1996 | 12.11 | 12.11 | 36.595 | 36.589 | 2.806°W | 2.776°W | 165 | 170 | 3.0 | 2.3 | 21.6 | 2771 | 59854 | 432482 | 1 | 5 | 0.084 | 0.002 | 0.000 |
| 31 | 09.05.1996 | 6.24 | 6.24 | 36.846 | 36.886 | 1.869°W | 1.834°W | 315 | 320 | 3.0 | 2.3 | 18.0 | 5458 | 98244 | 709877 | 1 | 7 | 0.071 | 0.002 | 0.000 |
| 32 | 09.05.1996 | 9.05 | 9.05 | 37.096 | 37.143 | 1.791°W | 1.764°W | 277 | 282 | 3.1 | 1.4 | 19.4 | 5806 | 112636 | 495400 | 26 | 69 | 0.613 | 0.015 | 0.002 |
| 33 | 09.05.1996 | 12.05 | 12.05 | 37.200 | 37.254 | 1.661°W | 1.632°W | 620 | 611 | 2.6 | 2.8 | 21.0 | 6514 | 136794 | 1203302 | 26 | 76 | 0.556 | 0.013 | 0.002 |
| 34 | 09.05.1996 | 15.30 | 15.30 | 37.379 | 37.376 | 1.380°W | 1.446°W | 415 | 420 | 2.7 | 1.2 | 18.5 | 5821 | 107689 | 405976 | 47 | 250 | 2.322 | 0.056 | 0.009 |
| 35 | 10.05.1996 | 6.23 | 6.23 | 38.072 | 38.095 | 0.228°W | 0.173°W | 232 | 242 | 3.0 | 2.5 | 22.0 | 5486 | 120692 | 947912 | 47 | 781 | 6.471 | 0.157 | 0.026 |
| 36 | 10.05.1996 | 8.38 | 8.38 | 38.033 | 38.015 | 0.311°W | 0.331°W | 156 | 147 | 2.9 | 1.8 | 15.1 | 2705 | 40846 | 230976 | 15 | 131 | 3.207 | 0.078 | 0.013 |
| 37 | 10.05.1996 | 11.21 | 11.21 | 37.870 | 37.892 | 0.130°W | 0.185°W | 467 | 481 | 3.0 | 2.3 | 16.0 | 5404 | 86464 | 624759 | 41 | 236 | 2.729 | 0.066 | 0.011 |
| 38 | 10.05.1996 | 14.18 | 14.18 | 37.734 | 37.755 | 0.134°W | 0.082°W | 533 | 529 | 2.8 | 2.4 | 20.9 | 5122 | 107050 | 807136 | 5 | 9 | 0.084 | 0.002 | 0.000 |
| 39 | 11.05.1996 | 6.15 | 6.15 | 37.817 | 37.792 | 0.412°W | 0.415°W | 159 | 159 | 3.0 | 1.2 | 16.4 | 2762 | 45297 | 170765 | 32 | 803 | 17.728 | 0.429 | 0.072 |
| 41 | 11.05.1996 | 10.01 | 10.01 | 37.903 | 37.929 | 0.586°W | 0.578°W | 66 | 72 | 3.2 | 2.9 | 17.3 | 2966 | 51312 | 467482 | 3 | 31 | 0.604 | 0.015 | 0.002 |
| 42 | 11.05.1996 | 11.29 | 11.29 | 37.963 | 37.984 | 0.514°W | 0.498°W | 94 | 94 | 3.0 | 2.3 | 18.4 | 2706 | 49790 | 359768 | 1 | 15 | 0.301 | 0.007 | 0.001 |
| 43 | 11.05.1996 | 13.53 | 13.53 | 38.191 | 38.215 | 0.240°W | 0.238°W | 88 | 84 | 2.9 | 2.8 | 17.6 | 2673 | 47045 | 413827 | 1 | 5 | 0.106 | 0.003 | 0.000 |
| 50 | 13.05.1996 | 12.51 | 12.51 | 38.493 | 38.448 | 0.478°E | 0.457°E | 296 | 313 | 2.9 | 2.6 | 21.1 | 5299 | 111809 | 913270 | 2 | 25 | 0.224 | 0.005 | 0.001 |
| 52 | 14.05.1996 | 6.32 | 6.32 | 38.830 | 38.872 | 0.841°E | 0.852°E | 668 | 676 | 2.6 | 2.6 | 22.0 | 4681 | 102982 | 841171 | 467 | 900 | 8.739 | 0.211 | 0.035 |
| 53 | 14.05.1996 | 8.46 | 8.46 | 38.883 | 38.928 | 0.922°E | 0.930°E | 600 | 595 | 2.8 | 2.6 | 21.7 | 5125 | 111213 | 908399 | 174 | 574 | 5.161 | 0.125 | 0.021 |
| 54 | 14.05.1996 | 11.25 | 11.25 | 38.882 | 38.840 | 0.971°E | 0.984°E | 467 | 471 | 2.6 | 2.3 | 18.0 | 4787 | 86166 | 622606 | 85 | 590 | 6.847 | 0.166 | 0.028 |
| 55 | 14.05.1996 | 13.09 | 13.09 | 38.858 | 38.906 | 1.026°E | 1.012°E | 281 | 275 | 3.0 | 2.6 | 18.3 | 5427 | 99314 | 811211 | 36 | 170 | 1.712 | 0.041 | 0.007 |
| 56 | 15.05.1996 | 6.14 | 6.14 | 39.896 | 39.914 | 0.894°E | 0.914°E | 144 | 146 | 3.0 | 2.4 | 18.4 | 2731 | 50250 | 378879 | 449 | 2500 | 49.751 | 1.204 | 0.201 |
| 57 | 15.05.1996 | 8.05 | 8.05 | 39.852 | 39.836 | 0.737°E | 0.712°E | 93 | 90 | 3.0 | 2.8 | 16.0 | 2730 | 43680 | 384229 | 44 | 285 | 6.525 | 0.158 | 0.026 |
| 58 | 15.05.1996 | 10.25 | 10.25 | 40.022 | 40.042 | 0.889°E | 0.910°E | 88 | 90 | 3.2 | 2.8 | 15.7 | 2916 | 45781 | 402712 | 2 | 5 | 0.109 | 0.003 | 0.000 |
| 59 | 15.05.1996 | 11.41 | 11.41 | 40.035 | 40.037 | 0.984°E | 1.021°E | 106 | 106 | 3.0 | 2.7 | 17.5 | 2761 | 48318 | 409843 | 23 | 261 | 5.402 | 0.131 | 0.022 |
| 60 | 15.05.1996 | 13.00 | 13.00 | 40.099 | 40.098 | 0.921°E | 0.887°E | 90 | 91 | 3.2 | 2.6 | 14.5 | 2947 | 42732 | 349037 | 4 | 18 | 0.421 | 0.010 | 0.002 |
| 62 | 16.05.1996 | 6.23 | 6.23 | 40.670 | 40.627 | 1.397°E | 1.385°E | 369 | 370 | 2.8 | 2.7 | 21.8 | 4954 | 107997 | 916064 | 56 | 220 | 2.037 | 0.049 | 0.008 |
| 63 | 16.05.1996 | 10.12 | 10.12 | 40.875 | 40.907 | 1.456°E | 1.507°E | 533 | 523 | 3.1 | 1.7 | 17.2 | 5559 | 95615 | 510650 | 17 | 48 | 0.502 | 0.012 | 0.002 |
| 64 | 16.05.1996 | 12.50 | 12.50 | 40.892 | 40.875 | 1.536°E | 1.483°E | 610 | 611 | 2.7 | 2.8 | 22.7 | 4855 | 110209 | 969444 | 49 | 126 | 1.143 | 0.028 | 0.005 |
| 67 | 17.05.1996 | 16.28 | 16.28 | 41.104 | 41.103 | 1.520°E | 1.551°E | 73 | 79 | 2.9 | 2.6 | 14.2 | 2638 | 37460 | 305975 | 1 | 7 | 0.187 | 0.005 | 0.001 |
| 73 | 19.05.1996 | 6.25 | 6.25 | 41.985 | 41.948 | 3.510°E | 3.476°E | 302 | 301 | 2.7 | 2.1 | 17.3 | 4979 | 86137 | 568273 | 9 | 38 | 0.441 | 0.011 | 0.002 |
| 74 | 19.05.1996 | 8.43 | 8.43 | 41.960 | 41.914 | 3.523°E | 3.509°E | 430 | 433 | 2.8 | 1.7 | 22.2 | 5211 | 115684 | 617835 | 102 | 307 | 2.654 | 0.064 | 0.011 |
| 75 | 19.05.1996 | 12.01 | 12.01 | 41.971 | 41.931 | 3.571°E | 3.549°E | 704 | 702 | 2.8 | 2.4 | 23.1 | 4809 | 111088 | 837582 | 11 | 25 | 0.225 | 0.005 | 0.001 |
| 76 | 19.05.1996 | 14.58 | 14.58 | 41.735 | 41.717 | 3.379°E | 3.357°E | 178 | 172 | 2.9 | 2.6 | 18.3 | 2689 | 49209 | 401943 | 471 | 151 | 3.069 | 0.074 | 0.012 |
| 78 | 20.05.1996 | 6.31 | 6.31 | 41.393 | 41.396 | 3.270°E | 3.211°E | 589 | 571 | 2.7 | 2.6 | 18.1 | 4931 | 89251 | 729015 | 51 | 180 | 2.017 | 0.049 | 0.008 |
| 79 | 20.05.1996 | 8.37 | 8.37 | 41.418 | 41.448 | 3.259°E | 3.298°E | 542 | 550 | 2.8 | 2.4 | 18.0 | 4688 | 84384 | 636240 | 25 | 97 | 1.150 | 0.028 | 0.005 |
| 80 | 20.05.1996 | 11.12 | 11.12 | 41.469 | 41.494 | 3.149°E | 3.089°E | 345 | 352 | 3.1 | 2.4 | 17.8 | 5659 | 100730 | 759487 | 34 | 127 | 1.261 | 0.031 | 0.005 |
| 83 | 21.05.1996 | 6.27 | 6.27 | 41.298 | 41.297 | 2.729°E | 2.693°E | 653 | 661 | 3.3 | 2.0 | 17.0 | 3047 | 51799 | 325462 | 5 | 15 | 0.290 | 0.007 | 0.001 |
| 105 | 27.05.1996 | 5.37 | 5.37 | 39.001 | 38.968 | 0.500°E | 0.538°E | 791 | 798 | 2.6 | 2.4 | 20.2 | 4935 | 99687 | 751622 | 19 | 65 | 0.652 | 0.016 | 0.003 |
| 106 | 27.05.1996 | 9.00 | 9.00 | 38.991 | 39.021 | 0.189°E | 0.184°E | 113 | 116 | 3.1 | 2.3 | 17.9 | 2832 | 50693 | 366289 | 1 | 1 | 0.020 | 0.000 | 0.000 |
|  |  |  |  |  |  |  |  |  |  |  |  |  |  |  |  |  |  |  |  |  |
| 53 | 24.05.1997 | 6.43 | 6.43 | 38.839 | 38.879 | 0.845°E | 0.868°E | 657 | 663 | 2.7 | 2.5 | 21.2 | 4876 | 103371 | 811875 | 7 | 12 | 0.116 | 0.003 | 0.000 |
| 54 | 24.05.1997 | 10.32 | 10.32 | 38.891 | 38.933 | 0.924°E | 0.929°E | 589 | 585 | 2.5 | 3.9 | 21.3 | 4656 | 99173 | 1215085 | 18 | 35 | 0.353 | 0.009 | 0.001 |
| 56 | 24.05.1997 | 15.19 | 15.19 | 38.901 | 38.859 | 1.010°E | 1.028°E | 293 | 253 | 2.7 | 3.5 | 20.0 | 4937 | 98740 | 1085702 | 2 | 6 | 0.061 | 0.001 | 0.000 |
| 68 | 27.05.1997 | 10.00 | 10.00 | 42.394 | 42.387 | 3.432°E | 3.400°E | 141 | 136 | 3.0 | 3.5 | 17.6 | 2760 | 48576 | 534121 | 1 | 6 | 0.124 | 0.003 | 0.000 |
| 73 | 28.05.1997 | 6.02 | 6.02 | 41.921 | 41.945 | 3.542°E | 3.582°E | 682 | 692 | 2.4 | 3.1 | 22.6 | 4236 | 95734 | 932343 | 1 | 2 | 0.021 | 0.001 | 0.000 |
| 74 | 28.05.1997 | 8.22 | 8.22 | 41.953 | 41.912 | 3.522°E | 3.502°E | 409 | 416 | 2.6 | 3.2 | 17.5 | 4794 | 83895 | 843404 | 4 | 6 | 0.072 | 0.002 | 0.000 |
| 75 | 28.05.1997 | 11.14 | 11.14 | 41.981 | 41.943 | 3.502°E | 3.471°E | 316 | 302 | 2.7 | 2.7 | 15.8 | 5002 | 79032 | 670369 | 1 | 3 | 0.038 | 0.001 | 0.000 |
| 78 | 26.05.1997 | 6.35 | 6.35 | 41.554 | 41.533 | 2.804°E | 2.788°E | 158 | 134 | 2.9 | 3.8 | 18.0 | 2650 | 47700 | 569445 | 1 | 25 | 0.524 | 0.013 | 0.002 |
| 81 | 29.05.1997 | 14.44 | 14.44 | 41.395 | 41.394 | 3.220°E | 3.272°E | 580 | 590 | 2.4 | 3.5 | 22.0 | 4321 | 95062 | 1045260 | 15 | 51 | 0.536 | 0.013 | 0.002 |
| 102 | 03.06.1997 | 13.19 | 13.19 | 39.001 | 38.969 | 0.489°E | 0.522°E | 737 | 785 | 2.6 | 2.7 | 19.7 | 4838 | 95309 | 808435 | 1 | 5 | 0.052 | 0.001 | 0.000 |
|  |  |  |  |  |  |  |  |  |  |  |  |  |  |  |  |  |  |  |  |  |
| 6 | 05.05.1998 | 10.26 | 10.26 | 36.277 | 36.316 | 5.069°W | 5.033°W | 367 | 365 | 2.9 | 2.0 | 19.2 | 5366 | 103027 | 647338 | 4 | 242 | 2.349 | 0.057 | 0.009 |
| 11 | 07.05.1998 | 5.34 | 5.34 | 36.395 | 36.389 | 4.478°W | 4.530°W | 562 | 570 | 2.6 | 1.4 | 16.5 | 4692 | 77418 | 340502 | 1 | 73 | 0.943 | 0.023 | 0.004 |
| 12 | 07.05.1998 | 8.19 | 8.19 | 36.364 | 36.359 | 4.422°W | 4.344°W | 660 | 671 | 3.8 | 2.2 | 20.1 | 6968 | 140057 | 968002 | 1 | 8 | 0.057 | 0.001 | 0.000 |
| 14 | 07.05.1998 | 14.36 | 14.36 | 36.354 | 36.364 | 4.720°W | 4.676°W | 558 | 559 | 2.2 | 2.5 | 22.2 | 4062 | 90176 | 708243 | 1 | 56 | 0.621 | 0.015 | 0.003 |
| 19 | 09.05.1998 | 3.15 | 3.15 | 36.569 | 36.540 | 3.072°W | 3.032°W | 584 | 579 | 2.6 | 2.5 | 22.4 | 4807 | 107677 | 845691 | 1 | 32 | 0.297 | 0.007 | 0.001 |
| 48 | 18.05.1998 | 15.05 | 15.05 | 38.472 | 38.430 | 0.469°E | 0.442°E | 301 | 311 | 2.8 | 3.4 | 18.3 | 5258 | 96221 | 1027780 | 5 | 13 | 0.135 | 0.003 | 0.001 |
|  |  |  |  |  |  |  |  |  |  |  |  |  |  |  |  |  |  |  |  |  |
| 32 | 12.05.1999 | 10.06 | 10.06 | 36.824 | 36.850 | 1.901°W | 1.852°W | 263 | 272 | 2.8 | 2.9 | 18.1 | 5241 | 94862 | 864252 | 1 | 2 | 0.021 | 0.001 | 0.000 |
| 47 | 16.05.1999 | 13.27 | 13.27 | 37.990 | 37.972 | 0.494°W | 0.509°W | 92 | 93 | 2.6 | 4.0 | 14.8 | 2323 | 34380 | 432036 | 1 | 3 | 0.087 | 0.002 | 0.000 |
| 56 | 19.05.1999 | 10.05 | 10.05 | 38.542 | 38.594 | 0.497°E | 0.503°E | 295 | 290 | 3.1 | 2.9 | 17.3 | 5786 | 100098 | 911952 | 1 | 48 | 0.480 | 0.012 | 0.002 |
| 81 | 25.05.1999 | 8.30 | 8.30 | 42.123 | 42.104 | 3.376°E | 3.356°E | 123 | 120 | 3.5 | 2.9 | 16.7 | 3166 | 52872 | 481698 | 1 | 84 | 1.589 | 0.038 | 0.006 |
|  |  |  |  |  |  |  |  |  |  |  |  |  |  |  |  |  |  |  |  |  |
| 6 | 23.05.2000 | 13.21 | 13.21 | 36.344 | 36.358 | 4.830°W | 4.779°W | 428 | 422 | 2.6 | 1.8 | 21.6 | 4848 | 104717 | 592159 | 1 | 20 | 0.191 | 0.005 | 0.001 |
| 10 | 24.05.2000 | 14.05 | 14.05 | 36.348 | 36.369 | 5.210°W | 5.201°W | 48 | 41 | 2.7 | 3.3 | 15.2 | 2496 | 37939 | 393325 | 1 | 56 | 1.476 | 0.036 | 0.006 |
| 19 | 26.05.2000 | 15.10 | 15.10 | 36.719 | 36.710 | 3.623°W | 3.594°W | 83 | 75 | 2.9 | 2.9 | 16.3 | 2763 | 45037 | 410314 | 1 | 9 | 0.200 | 0.005 | 0.001 |
| 36 | 31.05.2000 | 10.25 | 10.25 | 37.106 | 37.145 | 1.777°W | 1.747°W | 370 | 375 | 2.8 | 3.2 | 19.6 | 5170 | 101332 | 1018700 | 2 | 14 | 0.138 | 0.003 | 0.001 |
| 84 | 16.06.2000 | 13.35 | 13.35 | 42.401 | 42.395 | 3.437°E | 3.408°E | 139 | 134 | 2.7 | 3.3 | 17.3 | 2470 | 42731 | 443003 | 1 | 6 | 0.140 | 0.003 | 0.001 |
| 91 | 18.06.2000 | 13.08 | 13.08 | 41.413 | 41.393 | 2.749°E | 2.692°E | 269 | 273 | 2.8 | 3.2 | 19.2 | 5224 | 100301 | 1008333 | 1 | 3 | 0.030 | 0.001 | 0.000 |
|  |  |  |  |  |  |  |  |  |  |  |  |  |  |  |  |  |  |  |  |  |
| 5 | 13.05.2001 | 8.10 | 8.10 | 36.261 | 36.236 | 4.942°W | 4.993°W | 631 | 652 | 2.9 | 3.1 | 21.6 | 5374 | 116078 | 1130479 | 1 | 13 | 0.112 | 0.003 | 0.000 |
| 11 | 14.05.2001 | 11.55 | 11.55 | 36.368 | 36.370 | 4.516°W | 4.461°W | 652 | 651 | 2.7 | 3.2 | 22.6 | 4984 | 112638 | 1132364 | 1 | 1 | 0.009 | 0.000 | 0.000 |
| 18 | 17.05.2001 | 6.30 | 6.30 | 36.601 | 36.605 | 3.590°W | 3.540°W | 535 | 541 | 2.5 | 2.5 | 22.9 | 4540 | 103966 | 816546 | 1 | 4 | 0.038 | 0.001 | 0.000 |
| 30 | 20.05.2001 | 6.35 | 6.35 | 36.505 | 36.522 | 2.199°W | 2.150°W | 566 | 564 | 2.6 | 3.9 | 22.4 | 4789 | 107274 | 1314338 | 1 | 3 | 0.028 | 0.001 | 0.000 |
| 32 | 20.05.2001 | 12.54 | 12.54 | 36.824 | 36.860 | 1.784°W | 1.753°W | 725 | 715 | 2.6 | 4.0 | 22.6 | 4843 | 109452 | 1375411 | 1 | 1 | 0.009 | 0.000 | 0.000 |
| 41 | 23.05.2001 | 6.37 | 6.37 | 37.704 | 37.747 | 0.245°W | 0.229°W | 419 | 411 | 2.7 | 3.6 | 19.5 | 4971 | 96935 | 1096302 | 10 | 22 | 0.227 | 0.005 | 0.001 |
| 42 | 23.05.2001 | 8.35 | 8.35 | 37.732 | 37.747 | 0.152°W | 0.099°W | 523 | 528 | 2.7 | 3.3 | 22.4 | 4980 | 111552 | 1156487 | 9 | 18 | 0.161 | 0.004 | 0.001 |
| 43 | 23.05.2001 | 11.28 | 11.28 | 37.872 | 37.888 | 0.150°W | 0.201°W | 459 | 468 | 2.6 | 3.7 | 19.1 | 4793 | 91546 | 1064123 | 3 | 1 | 0.011 | 0.000 | 0.000 |
| 44 | 23.05.2001 | 13.33 | 13.33 | 37.875 | 37.909 | 0.287°W | 0.321°W | 403 | 393 | 2.6 | 3.5 | 18.6 | 4811 | 89485 | 983934 | 22 | 34 | 0.380 | 0.009 | 0.002 |
| 51 | 25.05.2001 | 5.29 | 5.29 | 38.336 | 38.373 | 0.470°E | 0.502°E | 576 | 569 | 2.6 | 3.8 | 21.1 | 4936 | 104150 | 1243342 | 2 | 6 | 0.058 | 0.001 | 0.000 |
| 55 | 27.05.2001 | 6.16 | 6.16 | 38.449 | 38.469 | 0.408°E | 0.421°E | 178 | 173 | 2.8 | 3.5 | 17.0 | 2603 | 44251 | 486565 | 1 | 4 | 0.090 | 0.002 | 0.000 |
| 63 | 28.05.2001 | 10.23 | 10.23 | 39.047 | 39.085 | 0.308°E | 0.270°E | 291 | 306 | 2.9 | 3.0 | 18.1 | 5381 | 97396 | 917936 | 1 | 1 | 0.010 | 0.000 | 0.000 |
| 64 | 28.05.2001 | 13.35 | 13.35 | 39.006 | 38.972 | 0.478°E | 0.582°E | 740 | 750 | 2.9 | 3.3 | 24.6 | 5339 | 131339 | 1361628 | 12 | 35 | 0.266 | 0.006 | 0.001 |
| 69 | 30.05.2001 | 7.54 | 7.54 | 38.693 | 38.641 | 1.063°E | 1.068°E | 214 | 216 | 3.0 | 3.5 | 19.2 | 5777 | 110918 | 1219610 | 1 | 4 | 0.036 | 0.001 | 0.000 |
| 71 | 01.06.2001 | 10.35 | 10.35 | 39.212 | 39.207 | 1.575°E | 1.513°E | 309 | 315 | 2.9 | 3.4 | 20.7 | 5367 | 111097 | 1186671 | 1 | 2 | 0.018 | 0.000 | 0.000 |
| 85 | 04.06.2001 | 8.51 | 8.51 | 40.908 | 40.877 | 1.509°E | 1.462°E | 524 | 532 | 2.8 | 3.0 | 21.9 | 5227 | 114471 | 1078866 | 3 | 2 | 0.017 | 0.000 | 0.000 |
| 86 | 04.06.2001 | 11.36 | 11.36 | 40.943 | 40.916 | 1.539°E | 1.492°E | 460 | 470 | 2.7 | 3.2 | 21.6 | 5002 | 108043 | 1086168 | 2 | 2 | 0.019 | 0.000 | 0.000 |
| 106 | 10.06.2001 | 6.42 | 6.42 | 41.392 | 41.391 | 3.297°E | 3.230°E | 612 | 616 | 3.0 | 3.0 | 22.7 | 5655 | 128369 | 1209844 | 1 | 1 | 0.008 | 0.000 | 0.000 |
| 107 | 10.06.2001 | 10.29 | 10.29 | 41.416 | 41.447 | 3.251°E | 3.295°E | 481 | 471 | 2.7 | 2.9 | 20.9 | 5068 | 105921 | 965007 | 24 | 10 | 0.094 | 0.002 | 0.000 |
| 108 | 10.06.2001 | 12.22 | 12.22 | 41.467 | 41.482 | 3.158°E | 3.098°E | 348 | 341 | 2.8 | 3.0 | 20.2 | 5269 | 106434 | 1003114 | 2 | 4 | 0.038 | 0.001 | 0.000 |
|  |  |  |  |  |  |  |  |  |  |  |  |  |  |  |  |  |  |  |  |  |
| 10 | 14.05.2002 | 6.15 | 6.15 | 36.350 | 36.371 | 5.209°W | 5.200°W | 50 | 34 | 2.4 | 3.5 | 12.5 | 2404 | 30050 | 330417 | 1 | 141 | 4.692 | 0.114 | 0.019 |
| 21 | 16.05.2002 | 11.24 | 11.24 | 36.706 | 36.719 | 3.583°W | 3.608°W | 67 | 68 | 2.8 | 2.9 | 16.8 | 2637 | 44302 | 403615 | 3 | 180 | 4.063 | 0.098 | 0.016 |
| 22 | 16.05.2002 | 13.54 | 13.54 | 36.683 | 36.688 | 3.275°W | 3.217°W | 371 | 386 | 2.8 | 2.4 | 20.3 | 5170 | 104951 | 791311 | 2 | 135 | 1.286 | 0.031 | 0.005 |
| 23 | 16.05.2002 | 15.35 | 15.35 | 36.663 | 36.663 | 3.216°W | 3.159°W | 638 | 592 | 2.8 | 2.1 | 23.2 | 5155 | 119596 | 789015 | 1 | 29 | 0.242 | 0.006 | 0.001 |
| 24 | 17.05.2002 | 6.26 | 6.26 | 36.595 | 36.591 | 2.817°W | 2.788°W | 178 | 181 | 2.9 | 2.4 | 18.8 | 2687 | 50516 | 380878 | 1 | 7 | 0.139 | 0.003 | 0.001 |
| 34 | 19.05.2002 | 13.56 | 13.56 | 36.484 | 36.479 | 2.926°W | 2.983°W | 602 | 622 | 2.8 | 2.6 | 22.5 | 5125 | 115313 | 941888 | 2 | 35 | 0.304 | 0.007 | 0.001 |
| 50 | 23.05.2002 | 15.22 | 15.22 | 37.875 | 37.895 | 0.283°W | 0.313°W | 414 | 330 | 2.9 | 1.9 | 20.0 | 4691 | 93820 | 560014 | 1 | 1 | 0.011 | 0.000 | 0.000 |
| 58 | 26.05.2002 | 11.37 | 11.37 | 38.070 | 38.099 | 0.225°W | 0.172°W | 208 | 220 | 3.0 | 2.9 | 16.4 | 5591 | 91692 | 835374 | 2 | 14 | 0.153 | 0.004 | 0.001 |
| 62 | 27.05.2002 | 11.40 | 11.40 | 38.337 | 38.374 | 0.470°E | 0.504°E | 545 | 576 | 2.7 | 2.8 | 22.7 | 5053 | 114703 | 1008980 | 3 | 6 | 0.052 | 0.001 | 0.000 |
| 63 | 27.05.2002 | 13.46 | 13.46 | 38.440 | 38.484 | 0.447°E | 0.477°E | 302 | 311 | 3.0 | 2.4 | 21.5 | 5538 | 119067 | 897743 | 1 | 2 | 0.017 | 0.000 | 0.000 |
| 72 | 29.05.2002 | 8.30 | 8.30 | 38.880 | 38.834 | 0.973°E | 0.986°E | 468 | 463 | 2.8 | 2.9 | 22.2 | 5240 | 116328 | 1059819 | 2 | 3 | 0.026 | 0.001 | 0.000 |
| 73 | 29.05.2002 | 12.00 | 12.00 | 38.833 | 38.879 | 0.838°E | 0.853°E | 662 | 704 | 2.8 | 2.4 | 23.4 | 5241 | 122639 | 924679 | 1 | 3 | 0.024 | 0.001 | 0.000 |
| 75 | 30.05.2002 | 6.29 | 6.29 | 39.630 | 39.591 | 0.320°E | 0.281°E | 305 | 329 | 2.9 | 2.9 | 20.6 | 5377 | 110766 | 1009148 | 1 | 2 | 0.018 | 0.000 | 0.000 |
| 78 | 30.05.2002 | 13.08 | 13.08 | 39.365 | 39.373 | 0.149°W | 0.180°W | 73 | 64 | 3.2 | 3.2 | 15.9 | 2826 | 44933 | 451719 | 1 | 4 | 0.089 | 0.002 | 0.000 |
| 98 | 04.06.2002 | 13.34 | 13.34 | 40.966 | 40.948 | 1.433°E | 1.409°E | 283 | 286 | 3.0 | 2.8 | 21.6 | 2840 | 61344 | 539610 | 5 | 5 | 0.082 | 0.002 | 0.000 |
| 100 | 05.06.2002 | 6.32 | 6.32 | 41.041 | 41.033 | 1.844°E | 1.780°E | 418 | 412 | 3.0 | 2.9 | 20.8 | 5481 | 114005 | 1038653 | 51 | 61 | 0.535 | 0.013 | 0.002 |
| 103 | 06.06.2002 | 8.43 | 8.43 | 41.297 | 41.306 | 2.261°E | 2.294°E | 154 | 151 | 3.4 | 3.2 | 17.6 | 2957 | 52043 | 523195 | 86 | 60 | 1.153 | 0.028 | 0.005 |
| 104 | 06.06.2002 | 11.35 | 11.35 | 41.283 | 41.291 | 2.320°E | 2.351°E | 224 | 227 | 2.8 | 3.1 | 19.0 | 2725 | 51775 | 504233 | 492 | 361 | 6.972 | 0.169 | 0.028 |
| 105 | 06.06.2002 | 13.17 | 13.17 | 41.218 | 41.211 | 2.326°E | 2.300°E | 402 | 398 | 2.4 | 1.2 | 23.6 | 4475 | 105610 | 398140 | 210 | 631 | 5.975 | 0.145 | 0.024 |
| 106 | 06.06.2002 | 15.40 | 15.40 | 41.169 | 41.176 | 2.413°E | 2.440°E | 702 | 770 | 2.6 | 3.0 | 23.1 | 4780 | 110418 | 1040664 | 175 | 233 | 2.110 | 0.051 | 0.009 |
| 108 | 07.06.2002 | 11.20 | 11.20 | 41.491 | 41.506 | 2.712°E | 2.739°E | 139 | 141 | 3.0 | 3.0 | 17.2 | 2809 | 48315 | 455356 | 1 | 2 | 0.041 | 0.001 | 0.000 |
| 110 | 08.06.2002 | 8.38 | 8.38 | 41.725 | 41.701 | 3.248°E | 3.234°E | 129 | 130 | 3.3 | 3.2 | 18.4 | 2961 | 54482 | 547716 | 271 | 407 | 7.470 | 0.181 | 0.030 |
| 111 | 08.06.2002 | 11.25 | 11.25 | 41.676 | 41.695 | 3.369°E | 3.386°E | 226 | 230 | 2.9 | 3.1 | 18.5 | 2616 | 48396 | 471325 | 299 | 1090 | 22.523 | 0.545 | 0.091 |
| 114 | 09.06.2002 | 11.15 | 11.15 | 41.980 | 41.958 | 3.273°E | 3.280°E | 112 | 131 | 2.7 | 3.4 | 18.6 | 2494 | 46388 | 495493 | 83 | 110 | 2.371 | 0.057 | 0.010 |
| 115 | 09.06.2002 | 13.26 | 13.26 | 41.947 | 41.966 | 3.471°E | 3.494°E | 293 | 314 | 3.2 | 2.6 | 19.8 | 5746 | 113771 | 929295 | 151 | 181 | 1.591 | 0.038 | 0.006 |
| 116 | 09.06.2002 | 15.28 | 15.28 | 41.949 | 41.900 | 3.526°E | 3.514°E | 430 | 480 | 2.9 | 2.5 | 20.3 | 5457 | 110777 | 870041 | 51 | 48 | 0.433 | 0.010 | 0.002 |
| 117 | 10.06.2002 | 6.31 | 6.31 | 41.388 | 41.390 | 3.301°E | 3.232°E | 638 | 617 | 3.1 | 2.5 | 23.1 | 5718 | 132086 | 1037399 | 121 | 149 | 1.128 | 0.027 | 0.005 |
| 118 | 10.06.2002 | 8.45 | 8.45 | 41.413 | 41.441 | 3.244°E | 3.288°E | 486 | 468 | 2.5 | 2.7 | 19.9 | 4762 | 94764 | 803814 | 159 | 317 | 3.345 | 0.081 | 0.014 |
| 119 | 10.06.2002 | 11.34 | 11.34 | 41.460 | 41.470 | 3.153°E | 3.122°E | 356 | 346 | 3.3 | 2.7 | 20.2 | 2828 | 57126 | 484556 | 1164 | 3200 | 56.017 | 1.355 | 0.226 |
| 120 | 10.06.2002 | 13.24 | 13.24 | 41.540 | 41.552 | 2.933°E | 2.901°E | 104 | 108 | 3.3 | 3.2 | 18.7 | 2994 | 55988 | 562850 | 52 | 50 | 0.893 | 0.022 | 0.004 |
|  |  |  |  |  |  |  |  |  |  |  |  |  |  |  |  |  |  |  |  |  |
| 4 | 27.04.2003 | 6.08 | 6.08 | 36.351 | 36.367 | 5.208°W | 5.183°W | 47 | 35 | 3.1 | 3.6 | 16.8 | 2800 | 47040 | 532009 | 3 | 394 | 8.376 | 0.203 | 0.034 |
| 6 | 27.04.2003 | 10.14 | 10.14 | 36.282 | 36.318 | 5.063°W | 5.022°W | 365 | 335 | 3.0 | 3.2 | 21.6 | 5376 | 116122 | 1167381 | 2 | 235 | 2.024 | 0.049 | 0.008 |
| 8 | 27.04.2003 | 15.00 | 15.00 | 36.289 | 36.299 | 4.791°W | 4.741°W | 753 | 761 | 2.5 | 3.1 | 24.2 | 4637 | 112215 | 1092858 | 1 | 4 | 0.036 | 0.001 | 0.000 |
| 10 | 28.04.2003 | 8.35 | 8.35 | 36.338 | 36.337 | 4.355°W | 4.303°W | 719 | 720 | 2.5 | 3.1 | 23.8 | 4655 | 110789 | 1078966 | 1 | 1 | 0.009 | 0.000 | 0.000 |
| 11 | 28.04.2003 | 11.53 | 11.53 | 36.528 | 36.553 | 4.388°W | 4.340°W | 257 | 252 | 2.8 | 3.1 | 20.1 | 5059 | 101686 | 990312 | 1 | 157 | 1.544 | 0.037 | 0.006 |
| 12 | 28.04.2003 | 13.43 | 13.43 | 36.565 | 36.588 | 4.267°W | 4.218°W | 304 | 308 | 2.7 | 2.9 | 20.2 | 5052 | 102050 | 929741 | 6 | 730 | 7.153 | 0.173 | 0.029 |
| 13 | 29.04.2003 | 7.43 | 7.43 | 36.616 | 36.628 | 4.095°W | 4.041°W | 341 | 346 | 2.7 | 3.2 | 21.6 | 4993 | 107849 | 1084213 | 2 | 209 | 1.938 | 0.047 | 0.008 |
| 15 | 29.04.2003 | 12.55 | 12.55 | 36.616 | 36.606 | 3.770°W | 3.717°W | 504 | 518 | 2.6 | 2.8 | 20.7 | 4807 | 99505 | 875290 | 1 | 206 | 2.070 | 0.050 | 0.008 |
| 18 | 30.04.2003 | 7.40 | 7.40 | 36.680 | 36.688 | 3.280°W | 3.222°W | 387 | 392 | 2.8 | 3.0 | 21.2 | 5217 | 110600 | 1042383 | 1 | 3 | 0.027 | 0.001 | 0.000 |
| 23 | 01.05.2003 | 7.25 | 7.25 | 36.718 | 36.714 | 2.277°W | 2.306°W | 251 | 216 | 2.8 | 3.4 | 17.6 | 2606 | 45866 | 489909 | 2 | 2 | 0.044 | 0.001 | 0.000 |
| 24 | 01.05.2003 | 9.11 | 9.11 | 36.651 | 36.640 | 2.414°W | 2.471°W | 331 | 321 | 2.8 | 3.4 | 20.2 | 5230 | 105646 | 1128448 | 17 | 145 | 1.373 | 0.033 | 0.006 |
| 26 | 01.05.2003 | 15.20 | 15.20 | 36.502 | 36.519 | 2.208°W | 2.157°W | 514 | 605 | 2.7 | 3.3 | 23.4 | 4917 | 115058 | 1192833 | 2 | 7 | 0.061 | 0.001 | 0.000 |
| 27 | 02.05.2003 | 6.22 | 6.22 | 36.838 | 36.856 | 1.888°W | 1.870°W | 269 | 292 | 2.7 | 2.9 | 20.2 | 2566 | 51833 | 472232 | 3 | 5 | 0.096 | 0.002 | 0.000 |
| 28 | 02.05.2003 | 8.39 | 8.39 | 36.863 | 36.825 | 1.750°W | 1.784°W | 707 | 730 | 2.8 | 3.1 | 23.5 | 5191 | 121989 | 1188037 | 7 | 11 | 0.090 | 0.002 | 0.000 |
| 44 | 05.05.2003 | 13.27 | 13.27 | 38.077 | 38.101 | 0.004°W | 0.049°E | 610 | 600 | 2.9 | 2.9 | 22.8 | 4764 | 108619 | 989587 | 7 | 73 | 0.672 | 0.016 | 0.003 |
| 45 | 06.05.2003 | 6.12 | 6.12 | 38.277 | 38.293 | 0.156°E | 0.180°E | 151 | 149 | 3.0 | 3.2 | 18.5 | 2736 | 50616 | 508847 | 2 | 10 | 0.198 | 0.005 | 0.001 |
| 46 | 06.05.2003 | 8.01 | 8.01 | 38.241 | 38.281 | 0.293°E | 0.330°E | 449 | 457 | 3.0 | 2.0 | 21.8 | 5545 | 120881 | 759517 | 1 | 3 | 0.025 | 0.001 | 0.000 |
| 47 | 06.05.2003 | 11.10 | 11.10 | 38.337 | 38.371 | 0.465°E | 0.511°E | 537 | 603 | 3.0 | 2.9 | 23.0 | 5474 | 125902 | 1147044 | 66 | 324 | 2.573 | 0.062 | 0.010 |
| 48 | 06.05.2003 | 13.36 | 13.36 | 38.447 | 38.491 | 0.451°E | 0.479°E | 296 | 305 | 2.9 | 2.9 | 22.2 | 5431 | 120568 | 1098450 | 30 | 163 | 1.352 | 0.033 | 0.005 |
| 49 | 06.05.2003 | 15.42 | 15.42 | 38.461 | 38.441 | 0.417°E | 0.401°E | 176 | 177 | 2.9 | 3.2 | 18.2 | 2671 | 48612 | 488703 | 7 | 65 | 1.337 | 0.032 | 0.005 |
| 50 | 07.05.2003 | 6.14 | 6.14 | 39.109 | 39.127 | 0.113°E | 0.096°E | 122 | 124 | 2.9 | 3.4 | 17.0 | 2533 | 43061 | 459952 | 3 | 2 | 0.046 | 0.001 | 0.000 |
| 51 | 07.05.2003 | 7.50 | 7.50 | 39.144 | 39.116 | 0.180°E | 0.232°E | 308 | 332 | 3.0 | 3.1 | 20.8 | 5448 | 113318 | 1103600 | 2 | 7 | 0.062 | 0.001 | 0.000 |
| 52 | 07.05.2003 | 10.54 | 10.54 | 39.014 | 38.980 | 0.480°E | 0.522°E | 748 | 774 | 2.9 | 3.4 | 17.0 | 5336 | 90712 | 968932 | 11 | 27 | 0.298 | 0.007 | 0.001 |
| 53 | 07.05.2003 | 14.04 | 14.04 | 38.932 | 38.951 | 0.374°E | 0.357°E | 188 | 186 | 2.8 | 3.1 | 17.5 | 2595 | 45413 | 442269 | 31 | 119 | 2.620 | 0.063 | 0.011 |
| 54 | 07.05.2003 | 16.00 | 16.00 | 38.898 | 38.916 | 0.222°E | 0.200°E | 99 | 98 | 2.9 | 3.6 | 16.9 | 2731 | 46154 | 521988 | 1 | 24 | 0.520 | 0.013 | 0.002 |
| 55 | 08.05.2003 | 6.19 | 6.19 | 39.610 | 39.571 | 0.301°E | 0.261°E | 235 | 279 | 3.0 | 3.2 | 21.3 | 5562 | 118471 | 1190995 | 40 | 198 | 1.671 | 0.040 | 0.007 |
| 56 | 08.05.2003 | 8.50 | 8.50 | 39.498 | 39.451 | 0.256°E | 0.190°E | 556 | 612 | 4.0 | 2.6 | 22.5 | 7792 | 175320 | 1432037 | 63 | 345 | 1.968 | 0.048 | 0.008 |
| 57 | 08.05.2003 | 11.34 | 11.34 | 39.434 | 39.412 | 0.020°W | 0.033°W | 115 | 113 | 2.9 | 3.0 | 17.9 | 2682 | 48008 | 452462 | 6 | 20 | 0.417 | 0.010 | 0.002 |
| 58 | 08.05.2003 | 12.56 | 12.56 | 39.367 | 39.390 | 0.092°W | 0.102°W | 89 | 86 | 3.0 | 3.3 | 16.0 | 2784 | 44544 | 461799 | 1 | 4 | 0.090 | 0.002 | 0.000 |
| 68 | 11.05.2003 | 6.14 | 6.14 | 39.829 | 39.845 | 0.702°E | 0.727°E | 95 | 96 | 3.0 | 3.1 | 16.7 | 2777 | 46376 | 451652 | 2 | 8 | 0.173 | 0.004 | 0.001 |
| 69 | 11.05.2003 | 7.54 | 7.54 | 39.894 | 39.912 | 0.889°E | 0.913°E | 144 | 151 | 3.0 | 3.0 | 18.1 | 2820 | 51042 | 481059 | 21 | 64 | 1.254 | 0.030 | 0.005 |
| 70 | 11.05.2003 | 9.53 | 9.53 | 40.012 | 40.031 | 0.874°E | 0.897°E | 92 | 94 | 3.2 | 3.2 | 16.2 | 2935 | 47547 | 477994 | 58 | 367 | 7.719 | 0.187 | 0.031 |
| 71 | 11.05.2003 | 11.22 | 11.22 | 40.037 | 40.069 | 0.995°E | 1.021°E | 125 | 115 | 3.0 | 3.1 | 17.0 | 2820 | 47940 | 466884 | 12 | 53 | 1.106 | 0.027 | 0.004 |
| 72 | 11.05.2003 | 12.56 | 12.56 | 40.107 | 40.129 | 1.134°E | 1.152°E | 175 | 180 | 3.0 | 2.9 | 17.5 | 2814 | 49245 | 448652 | 8 | 21 | 0.426 | 0.010 | 0.002 |
| 73 | 12.05.2003 | 6.13 | 6.13 | 40.310 | 40.285 | 1.269°E | 1.263°E | 137 | 140 | 3.0 | 2.9 | 17.3 | 2799 | 48423 | 441160 | 33 | 160 | 3.304 | 0.080 | 0.013 |
| 74 | 12.05.2003 | 7.50 | 7.50 | 40.300 | 40.323 | 1.121°E | 1.133°E | 96 | 96 | 3.0 | 3.1 | 18.4 | 2797 | 51465 | 501212 | 8 | 28 | 0.544 | 0.013 | 0.002 |
| 75 | 12.05.2003 | 9.51 | 9.51 | 40.412 | 40.436 | 1.045°E | 1.057°E | 88 | 86 | 3.1 | 3.1 | 17.9 | 2866 | 51301 | 499621 | 6 | 10 | 0.195 | 0.005 | 0.001 |
| 76 | 12.05.2003 | 11.20 | 11.20 | 40.531 | 40.544 | 1.014°E | 1.040°E | 78 | 79 | 2.9 | 3.2 | 17.5 | 2681 | 46918 | 471666 | 1 | 27 | 0.575 | 0.014 | 0.002 |
| 77 | 12.05.2003 | 12.38 | 12.38 | 40.606 | 40.630 | 1.108°E | 1.107°E | 85 | 87 | 2.8 | 3.2 | 17.6 | 2650 | 46640 | 468876 | 18 | 133 | 2.852 | 0.069 | 0.012 |
| 78 | 12.05.2003 | 15.15 | 15.15 | 40.924 | 40.949 | 1.229°E | 1.223°E | 89 | 86 | 3.0 | 3.1 | 17.5 | 2821 | 49368 | 480787 | 47 | 248 | 5.024 | 0.122 | 0.020 |
| 79 | 13.05.2003 | 7.01 | 7.01 | 41.085 | 41.083 | 1.334°E | 1.367°E | 67 | 71 | 3.0 | 3.2 | 16.6 | 2756 | 45750 | 459925 | 4 | 15 | 0.328 | 0.008 | 0.001 |
| 80 | 13.05.2003 | 8.18 | 8.18 | 41.023 | 41.006 | 1.376°E | 1.352°E | 141 | 143 | 2.9 | 3.1 | 19.2 | 2799 | 53741 | 523378 | 73 | 356 | 6.624 | 0.160 | 0.027 |
| 81 | 13.05.2003 | 10.34 | 10.34 | 40.935 | 40.918 | 1.424°E | 1.404°E | 336 | 338 | 2.7 | 3.1 | 19.2 | 2536 | 48691 | 474200 | 58 | 108 | 2.218 | 0.054 | 0.009 |
| 82 | 13.05.2003 | 12.16 | 12.16 | 40.876 | 40.896 | 1.489°E | 1.541°E | 587 | 607 | 2.7 | 2.1 | 23.6 | 4918 | 116065 | 765719 | 165 | 65 | 0.560 | 0.014 | 0.002 |
| 83 | 13.05.2003 | 14.44 | 14.44 | 40.862 | 40.849 | 1.520°E | 1.468°E | 684 | 690 | 2.5 | 2.5 | 23.2 | 4578 | 106210 | 834168 | 61 | 130 | 1.224 | 0.030 | 0.005 |
| 84 | 14.05.2003 | 6.30 | 6.30 | 41.161 | 41.172 | 2.366°E | 2.429°E | 661 | 735 | 2.9 | 2.7 | 24.3 | 5427 | 131876 | 1118612 | 57 | 171 | 1.297 | 0.031 | 0.005 |
| 86 | 14.05.2003 | 11.07 | 11.07 | 41.283 | 41.289 | 2.312°E | 2.346°E | 221 | 230 | 3.1 | 3.0 | 19.8 | 2844 | 56311 | 530720 | 44 | 8 | 0.142 | 0.003 | 0.001 |
| 87 | 14.05.2003 | 12.32 | 12.32 | 41.306 | 41.298 | 2.293°E | 2.260°E | 151 | 150 | 3.1 | 2.9 | 19.1 | 2874 | 54893 | 500112 | 4 | 33 | 0.601 | 0.015 | 0.002 |
| 89 | 15.05.2003 | 9.14 | 9.14 | 41.429 | 41.430 | 2.499°E | 2.532°E | 84 | 94 | 2.9 | 3.1 | 16.6 | 2696 | 44754 | 435852 | 11 | 55 | 1.229 | 0.030 | 0.005 |
| 90 | 15.05.2003 | 11.09 | 11.09 | 41.398 | 41.420 | 2.702°E | 2.765°E | 275 | 275 | 3.1 | 2.7 | 22.0 | 5828 | 128216 | 1087566 | 23 | 99 | 0.772 | 0.019 | 0.003 |
| 91 | 15.05.2003 | 13.54 | 13.54 | 41.302 | 41.282 | 2.728°E | 2.778°E | 624 | 650 | 2.5 | 2.2 | 22.8 | 4741 | 108095 | 747097 | 9 | 31 | 0.287 | 0.007 | 0.001 |
| 92 | 16.05.2003 | 6.24 | 6.24 | 42.005 | 41.981 | 3.597°E | 3.596°E | 621 | 657 | 2.6 | 2.8 | 23.1 | 2791 | 64472 | 567126 | 31 | 84 | 1.303 | 0.032 | 0.005 |
| 93 | 16.05.2003 | 8.46 | 8.46 | 41.906 | 41.952 | 3.516°E | 3.526°E | 475 | 424 | 2.8 | 2.6 | 21.8 | 5162 | 112532 | 919173 | 300 | 700 | 6.220 | 0.151 | 0.025 |
| 94 | 16.05.2003 | 11.31 | 11.31 | 42.126 | 42.170 | 3.625°E | 3.641°E | 539 | 529 | 2.8 | 2.6 | 21.3 | 5153 | 109759 | 896525 | 200 | 600 | 5.467 | 0.132 | 0.022 |
| 95 | 16.05.2003 | 13.43 | 13.43 | 42.109 | 42.088 | 3.593°E | 3.577°E | 465 | 435 | 2.8 | 2.9 | 20.8 | 2657 | 55266 | 503503 | 232 | 800 | 14.476 | 0.350 | 0.058 |
| 96 | 16.05.2003 | 15.45 | 15.45 | 42.128 | 42.107 | 3.385°E | 3.366°E | 125 | 123 | 3.0 | 3.1 | 18.2 | 2757 | 50177 | 488674 | 54 | 124 | 2.471 | 0.060 | 0.010 |
| 97 | 17.05.2003 | 6.15 | 6.15 | 42.394 | 42.387 | 3.430°E | 3.397°E | 140 | 137 | 3.0 | 3.0 | 18.6 | 2805 | 52173 | 491719 | 6 | 30 | 0.575 | 0.014 | 0.002 |
| 100 | 17.05.2003 | 11.15 | 11.15 | 41.977 | 41.952 | 3.271°E | 3.280°E | 112 | 142 | 3.1 | 2.9 | 18.2 | 2894 | 52671 | 479863 | 2 | 31 | 0.589 | 0.014 | 0.002 |
| 101 | 17.05.2003 | 13.44 | 13.44 | 41.700 | 41.680 | 3.394°E | 3.374°E | 236 | 228 | 3.0 | 3.0 | 18.9 | 2801 | 52939 | 498937 | 308 | 1000 | 18.890 | 0.457 | 0.076 |
| 104 | 18.05.2003 | 9.04 | 9.04 | 41.729 | 41.706 | 3.252°E | 3.237°E | 129 | 129 | 3.0 | 3.1 | 18.8 | 2764 | 51963 | 506066 | 19 | 94 | 1.809 | 0.044 | 0.007 |
| 105 | 18.05.2003 | 11.40 | 11.40 | 41.434 | 41.419 | 3.277°E | 3.255°E | 466 | 475 | 2.7 | 1.8 | 20.9 | 2461 | 51435 | 290857 | 27 | 109 | 2.119 | 0.051 | 0.009 |
| 106 | 18.05.2003 | 13.21 | 13.21 | 41.460 | 41.472 | 3.147°E | 3.117°E | 357 | 344 | 3.0 | 2.9 | 21.3 | 2813 | 59917 | 545880 | 114 | 496 | 8.278 | 0.200 | 0.033 |
| 107 | 18.05.2003 | 15.10 | 15.10 | 41.536 | 41.546 | 2.942°E | 2.911°E | 104 | 106 | 3.0 | 3.1 | 16.7 | 2728 | 45558 | 443682 | 25 | 162 | 3.556 | 0.086 | 0.014 |
| 108 | 19.05.2003 | 6.15 | 6.15 | 41.143 | 41.141 | 1.782°E | 1.815°E | 63 | 69 | 3.0 | 3.2 | 14.6 | 2768 | 40413 | 406273 | 1 | 5 | 0.124 | 0.003 | 0.000 |
| 109 | 19.05.2003 | 7.37 | 7.37 | 41.183 | 41.173 | 1.891°E | 1.862°E | 43 | 41 | 2.9 | 3.4 | 14.8 | 2711 | 40123 | 428568 | 1 | 3 | 0.075 | 0.002 | 0.000 |
| 111 | 20.05.2003 | 7.06 | 7.06 | 41.262 | 41.247 | 2.142°E | 2.132°E | 76 | 75 | 2.0 | 3.1 | 18.1 | 1846 | 33413 | 325403 | 13 | 73 | 2.185 | 0.053 | 0.009 |
| 114 | 20.05.2003 | 13.39 | 13.39 | 41.006 | 40.981 | 1.260°E | 1.261°E | 82 | 95 | 3.0 | 3.2 | 18.3 | 2687 | 49172 | 494331 | 4 | 20 | 0.407 | 0.010 | 0.002 |
| 115 | 20.05.2003 | 14.57 | 14.57 | 40.990 | 40.965 | 1.187°E | 1.190°E | 74 | 79 | 3.0 | 3.2 | 18.0 | 2787 | 50166 | 504323 | 2 | 16 | 0.319 | 0.008 | 0.001 |
|  |  |  |  |  |  |  |  |  |  |  |  |  |  |  |  |  |  |  |  |  |
| 15 | 09.05.2004 | 11.49 | 11.49 | 36.616 | 36.600 | 3.834°W | 3.883°W | 530 | 534 | 3.0 | 2.1 | 21.1 | 4760 | 100436 | 662610 | 1 | 2 | 0.020 | 0.000 | 0.000 |
| 23 | 11.05.2004 | 8.33 | 8.33 | 36.719 | 36.713 | 2.283°W | 2.313°W | 251 | 246 | 2.9 | 2.6 | 16.2 | 2735 | 44307 | 361906 | 11 | 30 | 0.677 | 0.016 | 0.003 |
| 27 | 12.05.2004 | 6.10 | 6.10 | 36.878 | 36.837 | 1.845°W | 1.879°W | 327 | 309 | 2.9 | 2.7 | 11.2 | 5380 | 60256 | 511109 | 2 | 10 | 0.166 | 0.004 | 0.001 |
| 28 | 12.05.2004 | 8.48 | 8.48 | 36.863 | 36.895 | 1.750°W | 1.742°W | 706 | 698 | 2.9 | 3.4 | 17.5 | 4562 | 79835 | 852750 | 188 | 419 | 5.248 | 0.127 | 0.021 |
| 29 | 12.05.2004 | 11.30 | 11.30 | 37.112 | 37.158 | 1.779°W | 1.756°W | 300 | 293 | 3.0 | 2.4 | 15.5 | 5553 | 86072 | 648963 | 20 | 41 | 0.476 | 0.012 | 0.002 |
| 30 | 12.05.2004 | 13.45 | 13.45 | 37.225 | 37.262 | 1.667°W | 1.635°W | 501 | 547 | 2.9 | 2.4 | 18.3 | 5015 | 91775 | 691963 | 6 | 20 | 0.218 | 0.005 | 0.001 |
| 32 | 13.05.2004 | 7.44 | 7.44 | 37.359 | 37.347 | 1.557°W | 1.584°W | 150 | 157 | 3.0 | 2.4 | 16.2 | 2747 | 44501 | 335532 | 2 | 5 | 0.112 | 0.003 | 0.000 |
| 33 | 13.05.2004 | 9.37 | 9.37 | 37.381 | 37.389 | 1.433°W | 1.372°W | 490 | 414 | 2.9 | 2.2 | 22.3 | 5427 | 121022 | 836444 | 906 | 1450 | 11.981 | 0.290 | 0.048 |
| 34 | 13.05.2004 | 12.13 | 12.13 | 37.508 | 37.512 | 1.180°W | 1.150°W | 178 | 185 | 3.0 | 2.5 | 18.1 | 2735 | 49504 | 388799 | 12 | 15 | 0.303 | 0.007 | 0.001 |
| 37 | 14.05.2004 | 5.56 | 5.56 | 37.686 | 37.729 | 0.335°W | 0.309°W | 256 | 250 | 2.8 | 2.4 | 16.1 | 5284 | 85072 | 641430 | 234 | 748 | 8.793 | 0.213 | 0.035 |
| 38 | 14.05.2004 | 8.07 | 8.07 | 37.716 | 37.760 | 0.245°W | 0.226°W | 406 | 398 | 2.8 | 2.3 | 19.4 | 5192 | 100725 | 727803 | 107 | 203 | 2.015 | 0.049 | 0.008 |
| 39 | 14.05.2004 | 10.28 | 10.28 | 37.729 | 37.747 | 0.157°W | 0.098°W | 520 | 521 | 3.0 | 2.4 | 20.4 | 5511 | 112424 | 847659 | 43 | 101 | 0.898 | 0.022 | 0.004 |
| 40 | 14.05.2004 | 13.05 | 13.05 | 37.871 | 37.892 | 0.136°W | 0.195°W | 458 | 500 | 3.1 | 2.4 | 21.2 | 5650 | 119780 | 903119 | 112 | 341 | 2.847 | 0.069 | 0.011 |
| 43 | 15.05.2004 | 9.07 | 9.07 | 37.927 | 37.950 | 0.472°W | 0.457°W | 110 | 110 | 3.0 | 3.6 | 18.1 | 2835 | 51314 | 580342 | 19 | 76 | 1.481 | 0.036 | 0.006 |
| 44 | 15.05.2004 | 10.59 | 10.59 | 38.006 | 38.030 | 0.577°W | 0.566°W | 59 | 59 | 3.0 | 3.4 | 17.3 | 2789 | 48250 | 515375 | 5 | 8 | 0.166 | 0.004 | 0.001 |
| 47 | 16.05.2004 | 10.55 | 10.55 | 38.121 | 38.112 | 0.064°W | 0.094°W | 272 | 279 | 2.9 | 3.5 | 18.8 | 5493 | 103268 | 1135494 | 7 | 26 | 0.252 | 0.006 | 0.001 |
| 48 | 16.05.2004 | 12.26 | 12.26 | 38.101 | 38.086 | 0.172°W | 0.197°W | 222 | 217 | 3.0 | 3.4 | 19.3 | 2783 | 53712 | 573719 | 9 | 35 | 0.652 | 0.016 | 0.003 |
| 49 | 16.05.2004 | 14.45 | 14.45 | 38.075 | 38.101 | 0.005°W | 0.052°E | 609 | 602 | 3.1 | 2.3 | 17.8 | 5014 | 89249 | 644884 | 33 | 133 | 1.490 | 0.036 | 0.006 |
| 51 | 17.05.2004 | 8.00 | 8.00 | 38.243 | 38.284 | 0.294°E | 0.330°E | 448 | 454 | 3.0 | 2.4 | 18.8 | 5509 | 103569 | 780893 | 244 | 440 | 4.248 | 0.103 | 0.017 |
| 52 | 17.05.2004 | 10.29 | 10.29 | 38.333 | 38.372 | 0.463°E | 0.504°E | 539 | 578 | 3.1 | 2.4 | 18.7 | 5621 | 105113 | 792530 | 242 | 774 | 7.364 | 0.178 | 0.030 |
| 53 | 17.05.2004 | 12.53 | 12.53 | 38.451 | 38.472 | 0.408°E | 0.423°E | 174 | 172 | 3.0 | 2.4 | 19.0 | 2735 | 51965 | 391807 | 99 | 296 | 5.696 | 0.138 | 0.023 |
| 54 | 17.05.2004 | 14.46 | 14.46 | 38.641 | 38.665 | 0.404°E | 0.396°E | 118 | 115 | 3.0 | 2.5 | 17.4 | 2813 | 48946 | 384422 | 10 | 25 | 0.511 | 0.012 | 0.002 |
| 55 | 18.05.2004 | 6.43 | 6.43 | 38.828 | 38.875 | 0.830°E | 0.850°E | 677 | 701 | 3.0 | 2.3 | 19.6 | 5516 | 108114 | 781192 | 320 | 400 | 3.700 | 0.090 | 0.015 |
| 56 | 18.05.2004 | 9.13 | 9.13 | 38.900 | 38.951 | 0.925°E | 0.935°E | 577 | 601 | 3.1 | 2.3 | 18.7 | 5669 | 106010 | 765994 | 303 | 979 | 9.235 | 0.223 | 0.037 |
| 57 | 18.05.2004 | 11.39 | 11.39 | 38.882 | 38.835 | 0.972°E | 0.988°E | 463 | 460 | 2.9 | 2.2 | 19.2 | 5446 | 104563 | 722688 | 551 | 1013 | 9.688 | 0.234 | 0.039 |
| 58 | 18.05.2004 | 14.08 | 14.08 | 38.894 | 38.869 | 1.017°E | 1.022°E | 266 | 282 | 3.0 | 2.4 | 18.3 | 2793 | 51112 | 385374 | 152 | 394 | 7.709 | 0.187 | 0.031 |
| 60 | 19.05.2004 | 6.44 | 6.44 | 38.984 | 39.022 | 0.500°E | 0.455°E | 740 | 730 | 3.1 | 2.3 | 21.2 | 5702 | 120882 | 873455 | 31 | 110 | 0.910 | 0.022 | 0.004 |
| 61 | 19.05.2004 | 9.36 | 9.36 | 39.111 | 39.124 | 0.243°E | 0.216°E | 341 | 322 | 3.0 | 2.3 | 19.2 | 2805 | 53856 | 389145 | 14 | 40 | 0.743 | 0.018 | 0.003 |
| 67 | 20.05.2004 | 12.55 | 12.55 | 39.458 | 39.482 | 0.189°E | 0.241°E | 600 | 580 | 2.8 | 2.4 | 18.9 | 5237 | 98979 | 746286 | 47 | 66 | 0.667 | 0.016 | 0.003 |
| 68 | 20.05.2004 | 15.13 | 15.13 | 39.566 | 39.588 | 0.255°E | 0.275°E | 282 | 282 | 3.1 | 2.3 | 18.6 | 2893 | 53810 | 388811 | 46 | 66 | 1.227 | 0.030 | 0.005 |
| 70 | 21.05.2004 | 7.57 | 7.57 | 39.899 | 39.916 | 0.898°E | 0.922°E | 147 | 167 | 3.1 | 2.3 | 18.2 | 2839 | 51670 | 373348 | 34 | 40 | 0.774 | 0.019 | 0.003 |
| 75 | 22.05.2004 | 6.17 | 6.17 | 40.293 | 40.316 | 1.263°E | 1.268°E | 135 | 130 | 2.8 | 2.0 | 13.0 | 2646 | 34398 | 216129 | 3 | 9 | 0.262 | 0.006 | 0.001 |
| 76 | 22.05.2004 | 8.18 | 8.18 | 40.302 | 40.324 | 1.120°E | 1.133°E | 95 | 95 | 3.0 | 2.3 | 17.2 | 2716 | 46715 | 337548 | 17 | 53 | 1.135 | 0.027 | 0.005 |
| 84 | 23.05.2004 | 14.19 | 14.19 | 40.862 | 40.851 | 1.520°E | 1.455°E | 684 | 684 | 3.0 | 2.3 | 20.1 | 5572 | 111997 | 809253 | 3 | 5 | 0.045 | 0.001 | 0.000 |
| 85 | 24.05.2004 | 6.35 | 6.35 | 41.162 | 41.176 | 2.371°E | 2.430°E | 661 | 724 | 2.9 | 2.2 | 18.8 | 5203 | 97816 | 676058 | 20 | 15 | 0.153 | 0.004 | 0.001 |
| 86 | 24.05.2004 | 8.57 | 8.57 | 41.221 | 41.207 | 2.348°E | 2.288°E | 403 | 398 | 2.8 | 1.9 | 16.0 | 5283 | 84528 | 504549 | 1 | 2 | 0.024 | 0.001 | 0.000 |
| 89 | 25.05.2004 | 6.45 | 6.45 | 41.387 | 41.392 | 3.297°E | 3.231°E | 639 | 606 | 3.0 | 2.2 | 20.6 | 5512 | 113547 | 784781 | 6 | 7 | 0.062 | 0.001 | 0.000 |
| 90 | 25.05.2004 | 9.10 | 9.10 | 41.420 | 41.454 | 3.247°E | 3.292°E | 568 | 540 | 3.0 | 2.3 | 20.1 | 5270 | 105927 | 765392 | 11 | 15 | 0.142 | 0.003 | 0.001 |
| 100 | 28.05.2004 | 6.34 | 6.34 | 42.130 | 42.176 | 3.625°E | 3.645°E | 538 | 534 | 3.0 | 2.5 | 20.1 | 5423 | 109002 | 856101 | 3 | 6 | 0.055 | 0.001 | 0.000 |
| 107 | 29.05.2004 | 12.32 | 12.32 | 41.912 | 41.959 | 3.513°E | 3.525°E | 452 | 409 | 2.9 | 2.5 | 19.3 | 5386 | 103950 | 816419 | 30 | 6 | 0.058 | 0.001 | 0.000 |
| 108 | 29.05.2004 | 15.00 | 15.00 | 41.969 | 42.014 | 3.576°E | 3.612°E | 695 | 631 | 3.1 | 2.7 | 17.8 | 5763 | 102581 | 870125 | 3 | 5 | 0.049 | 0.001 | 0.000 |
| 122 | 02.06.2004 | 7.32 | 7.32 | 36.504 | 36.528 | 2.201°W | 2.148°W | 500 | 448 | 3.0 | 2.4 | 20.3 | 5437 | 110371 | 832178 | 1 | 4 | 0.036 | 0.001 | 0.000 |
|  |  |  |  |  |  |  |  |  |  |  |  |  |  |  |  |  |  |  |  |  |
| 36 | 18.05.2005 | 5.59 | 5.59 | 37.796 | 37.820 | 0.624°W | 0.935°E | 47 | 44 | 3.0 | 2.9 | 16.0 | 2841 | 45456 | 414132 | 1 | 2 | 0.044 | 0.001 | 0.000 |
| 54 | 22.05.2005 | 10.26 | 10.26 | 38.898 | 38.946 | 0.925°E | 0.733°E | 577 | 590 | 2.9 | 2.0 | 18.1 | 5333 | 96527 | 606498 | 2 | 26 | 0.269 | 0.007 | 0.001 |
| 67 | 25.05.2005 | 6.11 | 6.11 | 39.833 | 39.849 | 0.709°E | 0.919°E | 95 | 97 | 3.0 | 2.8 | 18.3 | 2790 | 51057 | 449120 | 4 | 86 | 1.684 | 0.041 | 0.007 |
| 68 | 25.05.2005 | 7.51 | 7.51 | 39.900 | 39.918 | 0.896°E | 1.133°E | 142 | 153 | 3.0 | 2.6 | 19.1 | 2751 | 52544 | 429187 | 3 | 53 | 1.009 | 0.024 | 0.004 |
| 74 | 26.05.2005 | 7.04 | 7.04 | 40.300 | 40.322 | 1.120°E | 2.723°E | 96 | 95 | 2.9 | 2.8 | 15.5 | 2704 | 41912 | 368677 | 10 | 206 | 4.915 | 0.119 | 0.020 |
| 89 | 30.05.2005 | 12.13 | 12.13 | 41.280 | 41.303 | 2.783°E | 3.554°E | 636 | 628 | 3.0 | 2.5 | 22.3 | 5549 | 123743 | 971872 | 1 | 4 | 0.032 | 0.001 | 0.000 |
