# Supplementary material for: Sinking Jelly-Carbon Unveils Potential Environmental Variability along a Continental Margin
Source: PLoS One. 2013 Dec 18;8(12):e82070. doi: 10.1371/journal.pone.0082070 (PMC3867349; doi:10.1371/journal.pone.0082070)
Supplement: Text S1 — Supplementary text about the methodology used. Discussion of the study caveats and comparisons with sediment trap data. (DOC) [file pone.0082070.s010.doc]

**Text S1**

**Study caveats**

We assumed that most pyrosomes collected by trawling were dead, although we acknowledge a strong daily vertical migration (down to 700 m wide) component and the accumulation of living and dead gelatinous zooplankton (evidenced by using opening/closing nets) close to the bottom on the slope, to 1200-1400 m. In both cases however, dead or living organisms accumulating, our results show consistent evidence on the significant increase of pyrosome biomass concurrent with hydroclimatic modifications in the northwestern Mediterranean. Moreover, our observations must be considered cautiously owing to methodological aspects such as the integration of large areas (30,050 to 175,320 m^2^), which mask local accumulations around benthic features that drastically increases the carbon per unit area.

**Comparisons of sediment trap vs. jelly-carbon fluxes**

Selective sediment trap observations (Fig. 3) in the vicinity of the three sectors were compared with our jelly-carbon (Table S2) deposits merely to have a reference (no intention to compare the jelly carbon and the particle carbon export per se). Jelly-carbon deposition data were calculated after seasonal cruises (3 months every year), which does not allow to calculate year/daily averages as conventionally done by sediment trap studies. Sediment trap daily fluxes comparison with our monthly figures is not possible owing to a methodological reason. Trap work is a much standardised science, where the collector mechanisms (trap, cup, etc) are place in the water column in a moored line at different depths and/or are buoyant traps travelling with the current. The data compared here originate in fixed mooring lines in the offshore, thus fluxes only reflect the carbon export at one place, which then is extrapolated to thousands of km^3^ of water. The trap allows to recover fluxes on a monthly basis during the course of a year and then to average daily rates with conversions. Our jelly-carbon depositions data use a radically different approach, which is sampling 10s of km^2^ of seabed to actually jelly-carbon direct deposits (and from one bloom-forming species). If the trap approach deployed 1000 traps in the area a different result will be obtained. Furthermore, traps collect sediments at mid-water (at least the ones in Table S2), while jelly-carbon deposits are collected directly from the seabed. Except when using submarines, video/photo cameras to assess the jelly-carbon transfer, there are no other methods apart from trawling to know how much carbon is transported. A trawling survey (10s of km^2^) and a sediment trap (place in a few cubic meters of water) cannot reproduce the same result or be actually comparable. A last issue is that we present only jelly-carbon depositions data from one species found in the trawls (Pyrosomes). This does not preclude other bloom-forming species to export carbon. It is also possible that trawling cannot sample for this additional jelly-biomass since it may actually remineralize in the water column. From a methodological perspective we conclude that trawling surveys serve to initially quantify jelly-carbon deposits at large scale but they may miss significant inputs that need to be quantify visually e.g. via ROVs or cameras. Ultimately, to provide a jelly-carbon vs. particulate-carbon comparison we need to treat both compartments with biogeochemical models to reduce uncertainties involved in the sampling process.
